# Optimization of a novel trapezoidal staggered ribs configuration for enhancement of a solar air heater performance using CFD

**DOI:** 10.1007/s11356-023-28978-9

**Published:** 2023-07-28

**Authors:** Sarvapriya Singh, Siddharth Suman, Santanu Mitra, Manish Kumar

**Affiliations:** 1 Department of Mechanical Engineering, Shiv Nadar Institute of Eminence, Tehsil Dadri, Uttar Pradesh 201314 India; 2 Centre for Nuclear Safety, VTT Technical Research Centre of Finland, 02150 Espoo, Finland; 3 Department of Mechanical Engineering, Malaviya National Institute of Technology, Jaipur, 302017 India

**Keywords:** Computational fluid dynamics, Heat transfer, Discontinuous ribs, Nusselt number, Friction factor, Energy

## Abstract

A novel transverse trapezoidal staggered ribs configuration as artificial roughness is investigated using CFD to understand the fluid flow and heat transfer behaviors for improving the performance of a solar air heater. In addition, experimental validation of Nusselt numbers for smooth duct against CFD results is established. The staggered ribs arrangement outperforms the continuous ribs and insights obtained from the thermal-fluid flow behaviors are further applied to optimize the staggered arrangements of the rib. Reynolds number *Re* is varied from 5000 to 24,000, and rib parameters are optimized with a special focus on understanding the effects of discontinuous rib width (*w*) and gap width (*g*). Two widely studied shapes of rib, namely, cylindrical and rectangular, are also investigated for the transverse staggered arrangement with the same optimized parameters for the comparison of thermo-hydraulic performances. Trapezoidal ribs having discontinuous rib width of 50 mm and gap width of 10 mm give the maximum thermo-hydraulic performance of 1.57 at Reynolds number of 5000. The optimized staggered trapezoidal ribs outperform the staggered cylindrical and rectangular ribs. However, staggered rectangular ribs give the highest increase in the Nusselt number and these may be preferred for application when pressure drop is not of concern.

**Supplementary Information:**

The online version contains supplementary material available at 10.1007/s11356-023-28978-9.

## Introduction

A solar air heater (SAH) is typically a heat exchanger that uses solar irradiation falling on the absorber or collector plate to raise the temperature of the air flowing in a rectangular duct at ambient temperature. SAHs have a wide range of uses, including space heating, grain drying, and timber seasoning, all of which require a low-temperature difference to achieve the requirements (Singh et al. 2022a, b). However, flat plate SAHs have poor thermal performance inhibiting their wide applicability (Singh et al. 2021). One of the prime reasons for poor thermal performance is the inherent properties of air—less heat absorbing capacity, low convective heat transfer coefficient, and the development of a viscous sublayer at the absorber plate interface in a fully developed turbulent flow. As a result of these drawbacks, the temperature of the absorber plate rises, causing more heat to be lost to the environment rather than convecting to the incoming air in the duct. In order to improve the thermal performance of the SAHs, a passive technique employing surface geometries on the underside surface of the absorber plate at the air interface is a popular method (Suman et al. 2015). Artificial roughness is generally employed to disrupt the production of viscous sublayer at the contact, resulting in improved heat transfer. Many researchers have looked into the effects of different continuous transverse rib configurations on the thermal performance of roughened SAHs. Since continuous ribs having uniform and non-uniform cross-sections are easy to fabricate and install, therefore, small diameter transverse protruding wires (Prasad and Mullick 1983; Prasad and Saini, 1988; Gupta et al. 1993), repeated chamfered (Karwa et al. 1999), thin circular wires (Verma and Prasad 2000), wedge-shaped (Bhagoria et al. 2002), thin circular wires in arc shape (Kumar and Saini 2009), transverse repeated chamfered-grooves (Layek et al. 2009), S-shaped (Kumar et al. 2017), multiple V-shaped (Jin et al. 2019), and saw-tooth (Singh et al. 2015) as roughened ribs have been investigated experimentally in the starting years of exploration in the application field of solar-thermal. All the investigated studies have reported that the performance of roughened ducts is enhanced at the expanse of pressure drop increase. Therefore, to reduce pressure drop and further enhance the thermo-hydraulic performance factor (THPF), transverse ribs with gaps are being explored by researchers primarily using experimental investigations. A summary of studies on discontinuous ribs is provided in Table 1. It is concluded from the research articles discussed in Table 1 that discontinuities in the ribs result in a higher heat transfer rate due to high-energy fluid streams formed through the gaps, thus creating more mixing with the less-energy fluid streams. The space provided in the discontinuous ribs also helps in reducing pressure loss because of lesser obstruction in the fluid domain. Cavallero and Tanda (2002) did an experimental investigation with the help of liquid crystal thermography (LCT) in a rectangular duct roughened with rectangular ribs arranged transversely in continuous and broken form. Their investigation reported that the broken ribs perform better in heat transfer coefficient than continuous ribs. An experimental investigation by Karwa (2003) also proved that the discrete ribs always perform better than the continuous ribs under similar operating conditions with the same geometric parameters. His investigation also indicated that the secondary flow generated in the discrete ribs was the main reason for enhancing the roughened duct’s thermal performance. Again, by Tanda (2004a), the thermal performance of roughened rectangular duct was evaluated with the help of the LCT technique by considering the transverse and V-shaped ribs in continuous and broken arrangement with square and rectangular cross-sections of the ribs. His investigation concluded that the broken ribs in any pattern mostly perform better due to the end-wall vortices generated. His findings also mentioned that the local heat transfer coefficient distribution between inter-ribs was mainly affected by the geometric cross-sections and configurations of the ribs. Discontinuous metal grits in the staggered arrangement at different angles having circular cross-sections were employed on collector plates and tested experimentally by Karmare and Tikekar (2007). They observed that the secondary flow near the duct walls enhanced the performance of the roughened rectangular duct. Aharwal et al. (2008) conducted an experimental investigation with the discontinuous inclined ribs having a square cross-section in a roughened rectangular duct for the fixed angle of attack. They concluded that secondary flow formed at the back of ribs in continuous transverse ribs accelerated the primary flow in discontinuous ribs and enhanced the local heat transfer coefficient near the end-wall regions. Singh et al. (2011) conducted an experiment with discrete V-down ribs to conclude the optimized rib dimensions. Their investigation emphasized the variation of gap width and gap position with *Re* for all other considered roughness parameters. They found that the optimum gap between ribs was required to increase the local heat transfer rate between the discrete ribs. Moreover, they also observed that secondary flow generated from the leading to the trailing edge was the main factor in boosting the local heat transfer rate. Kumar et al. (2013) conducted the experimental study with multi-V-shaped ribs with gaps created symmetrically to optimize the heat transfer and fluid flow friction in a roughened rectangular duct. They considered a wide range of roughness parameters and concluded the optimized results in non-dimensional parameters for *Nu* and *f*. They also noted that the secondary flow development enhanced the local heat transfer behind the discontinuous ribs in the downstream region. Again, the circular wire ribs were used to roughen the rectangular duct by Maithani and Saini (2016), arranged in a V-shaped pattern with symmetric gaps ranging from 1 to 4 for different roughness parameters along with Reynolds number. They reported that the *Nu* increases with an increase in *Re* till the number of gaps is equal to 3; after that, the heat transfer was decreased due to a decrease in the surface area of the roughened plate. The enhancement in the *Nu* was also observed on the higher side until the relative gap width equaled to 4. Gill et al. (2017a) conducted an experimental investigation with the circular wires attached to an absorber plate in the arc form with discontinuities combined with a staggered arrangement of the ribs in front of the gaps. They did observe that the *Nu* and *f* both increased with the relative staggered rib size increases from 1 to 4 after that, decreased with 5 and 6. It was observed that the gaps provided in the continuous ribs and ribs arranged in a staggered manner performed better than the continuous ribs without the staggered arrangement. The influence of gap position was studied by Gill et al. (2017b) with broken arc ribs on an absorber plate arranged symmetrically in a rectangular duct, and results were reported in terms of relative gap position. They observed that the gap provided in the continuous ribs resulted in higher thermal performance of roughened duct compared to the continuous ribs without the gaps under similar geometric and flow conditions. The reason was reported that the secondary flow developed in the upstream region of the continuous ribs was getting the space to mix with the primary flow. Thus, fluid was accelerated through the gaps resulting in higher local heat transfer. A comparative study was done by Singh et al. (2019a) to investigate the thermal performance of roughened duct with multiple broken transverse and square wave-shaped ribs on an absorber plate. They found that the gaps in the multiple transverse ribs performed better than the square wave-shaped and broken transverse ribs. This was attributed because of the accelerated flow through gaps which consequently increased the local heat transfer near the end walls of the broken ribs. They did observe that the pressure drop was calculated lower with the multiple transverse broken ribs than with the square wave-shaped ribs. Cylindrical ribs arranged in novel V-shaped with gaps combined with staggered ribs were studied by Singh Patel and Lanjewar (2019) experimentally and numerically to get an insight picture of fluid flow behavior and the reason behind the augmentation of the average Nusselt number. They concluded that the novel V-shaped ribs supported with staggered ribs perform better than the discontinuous ribs arranged in V-shaped without the staggered elements.

**Table 1:** Summary of studies on SAH with discontinuous ribs

Reference	Rib configurations	Range of geometric and flow parameters	Findings
Cavallero and Tanda (2002)	Rectangular (transverse continuous and broken)	*e/D*_*h*_ = 0.15, *P/e* = 4–8, *P/w* = 6.66–13.33, *L*_2_ = 280 mm, *W/H* = 5, *Re* = 8000–35,000	A broken rib with lower relative pitch roughness (*P/e* = 4) was found to have a maximum local heat transfer coefficient between the ribs
Karwa (2003)	Rectangular (repeated transverse, inclined, V-continuous, V-discrete)	*e/D*_*h*_ = 0.0467–0.05, *P/e* = 10, *W/H* = 7.19–7.75, *Re* = 2800–15,000, *α* = 60°	V-down repeated discrete ribs gave the highest *Nu* ratio and thermal performance factor
Tanda (2004a)	Rectangular and square (transverse continuous and broken, V-shaped broken)	*e/D*_*h*_ = 0.09–0.15, *P/e* = 4–13.3, *W/H* = 5, *Re* = 5000–50,000, *α* = 45–60°	A staggered transverse rectangular rib with *P/e* = 4 was the best in heat transfer augmentation
Karmare and Tikekar (2007)	Circular (broken metal grits arranged in staggered)	*e/D*_*h*_ = 0.035–0.044, *P/e* = 12.5–36, *l/s* = 1–1.72, *W/H* = 10, *Re* = 4000–17,000, *α* = 45–60°	Metal grit with *l/s* = 1.72, *e/D*_*h*_ = 0.044, and *P/e* = 17.5 yielded the highest heat transfer coefficient and was found to be optimum in terms of thermal performance
Aharwal et al. (2008)	Square (angled broken ribs)	*e/D*_*h*_ = 0.0377, *P/e* = 10, *g/e* = 0.5–2, *d/W* = 0.1667–0.667, *W/H* = 5.84, *Re* = 3000–18,000, *α* = 60°	An inclined broken rib with *d/W* = 0.25 and *g/e* = 1 yielded the maximum THPF
Singh et al. (2011)	Circular wires (discrete V-down ribs)	*e/D*_*h*_ = 0.015–0.043, *P/e* = 4–12, *g/e* = 0.5–2, *d/W* = 0.2–0.8, *W/H* = 12, *Re* = 3000–18,000, *α* = 30–75°	Discrete V-down ribs with *d/w* = 0.65, *g/e* = 1, *P/e* = 8, *α* = 60°, and *e/D*_*h*_ = 0.043 reported the maximum values of *Nu* for all the investigated *Re* and *f* after *Re* = 9000
Kumar et al. (2013)	Circular wires (multi-V-shaped with gaps ribs)	*e/D*_*h*_ = 0.022–0.043, *P/e* = 6–15, *G*_*d*_*/L*_*v*_ = 0.24–0.80, *W/w* = 1–10, *W/H* = 12, *Re* = 2000–20,000, *α* = 30–75°	The maximum enhancement in *Nu* occurred at *e/D*_*h*_ = 0.043, *G*_*d*_*/L*_*v*_ = 0.69, *g/e* = 1, *W/w* = 6, *P/e* = 8, and *α* = 30°
Maithani and Saini (2016)	Circular wires (symmetric discontinuous ribs)	*e/D*_*h*_ = 0.043, *P/e* = 6–12, *g/e* = 1–5, *N*_*g*_ = 1–5, *W/H* = 12, *Re* = 4000–18,000, *α* = 30–75°	The highest *Nu* and *f* were found with *g/e* = 3, *P/e* = 10, *N*_*g*_ = 3, and *α* = 60° for all the considered ranges of *Re*
Gill et al. (2017a)	Circular wires (broken ribs combined with staggered ribs)	*e/D*_*h*_ = 0.043, *P/e* = 10, *p′/P* = 0.4, *g/e* = 1, *r/g* = 1–6, *w′/w* = 0.65, *W/H* = 12, *Re* = 2000–16,000, *α* = 30°	Maximum THPF = 2.27 was found with *r/g* = 4 at *Re* = 12,000
Gill et al. (2017b)	Circular wires (broken arc ribs)	*e/D*_*h*_ = 0.043, *P/e* = 8, *g/e* = 1, *d/w* = 0.2–0.8, *W/H* = 12, *Re* = 2000–16,000, *α* = 30°	The highest THPF = 1.94 was yielded with *d/w* = 0.65 at *Re* = 12,000
Singh et al. (2019a)	Square ribs (multiple broken transverse ribs and square wave-shaped ribs)	*e/D*_*h*_ = 0.043, *P/e* = 8, *W/w* = 7, *W/H* = 12, *Re* = 3000–18,000, *α* = 30°	The highest THPF was 1.62 at *Re* = 15,000 for square wave-shaped ribs, while 2.10 was obtained at *Re* = 15,000 for multiple broken transverse ribs
Singh Patel and Lanjewar (2019)	Circular wires (multiple gaps V-shaped combined with staggered ribs)	*e/D*_*h*_ = 0.043, *P/e* = 6–14, *p′/P* = 0.4, *g/e* = 4, *g′/e* = 4, *r/e* = 4, *d/w* = 0.65, *N*_*g*_ = 3, *n*_*g*_ = 4, *W/H* = 12, *Re* = 4000–14,500, *α* = 60°	Maximum THPF was found to be 1.59 for *P/e* = 10 at *Re* = 12,364

A thorough survey of the literature revealed that even though many studies have been done with discontinuous ribs experimentally, there is no holistic understanding of heat and fluid flow behaviors that can be critical to optimize the design and thus maximize THPF. For example, the disadvantage reported with the continuous rib was that the re-circulation zones formed behind the continuous ribs were not able to mix with the primary flow and stuck behind the ribs, as predicted by the experimental tests. This problem could be easily eliminated with discontinuous ribs, potentially improving the roughened duct’s thermal performance. Hence, it is imperative to optimize the design using insights obtained from heat and fluid flow behaviors using numerical studies. As a result, the emphasis in the present work is placed on conducting a numerical analysis to gain insight into the thermal-fluid characteristics of discontinuous ribs. The following key objectives were set for the present investigation:

- The effects of gap width (*g*) and discontinuous rib width (*w*) provided in discontinuous ribs on fluid flow and heat transfer.
- Establishing a flow pattern that supports an increase in THPF.
- Effects of the discontinuous ribs arranged in a staggered configuration over the in-line configuration.

To achieve these objectives, artificially roughened ribs with different cross-sectionals are simulated. Additionally, a thermal performance comparison of continuous ribs with discontinuous ribs is presented in this research work. Furthermore, discontinuous ribs are arranged in staggered, and in-line configurations also investigated to gain an understanding of the impacts of the different configurations.

## Computational fluid dynamics (CFD) modelling

A steady three-dimensional incompressible fluid flows in a rectangular roughened duct solved numerically using commercial software ANSYS Fluent™ code 2021 R2 research license. The rectangular duct is roughened with transverse continuous and discontinuous ribs.

### Geometric details of the model

**Fig. 1:**
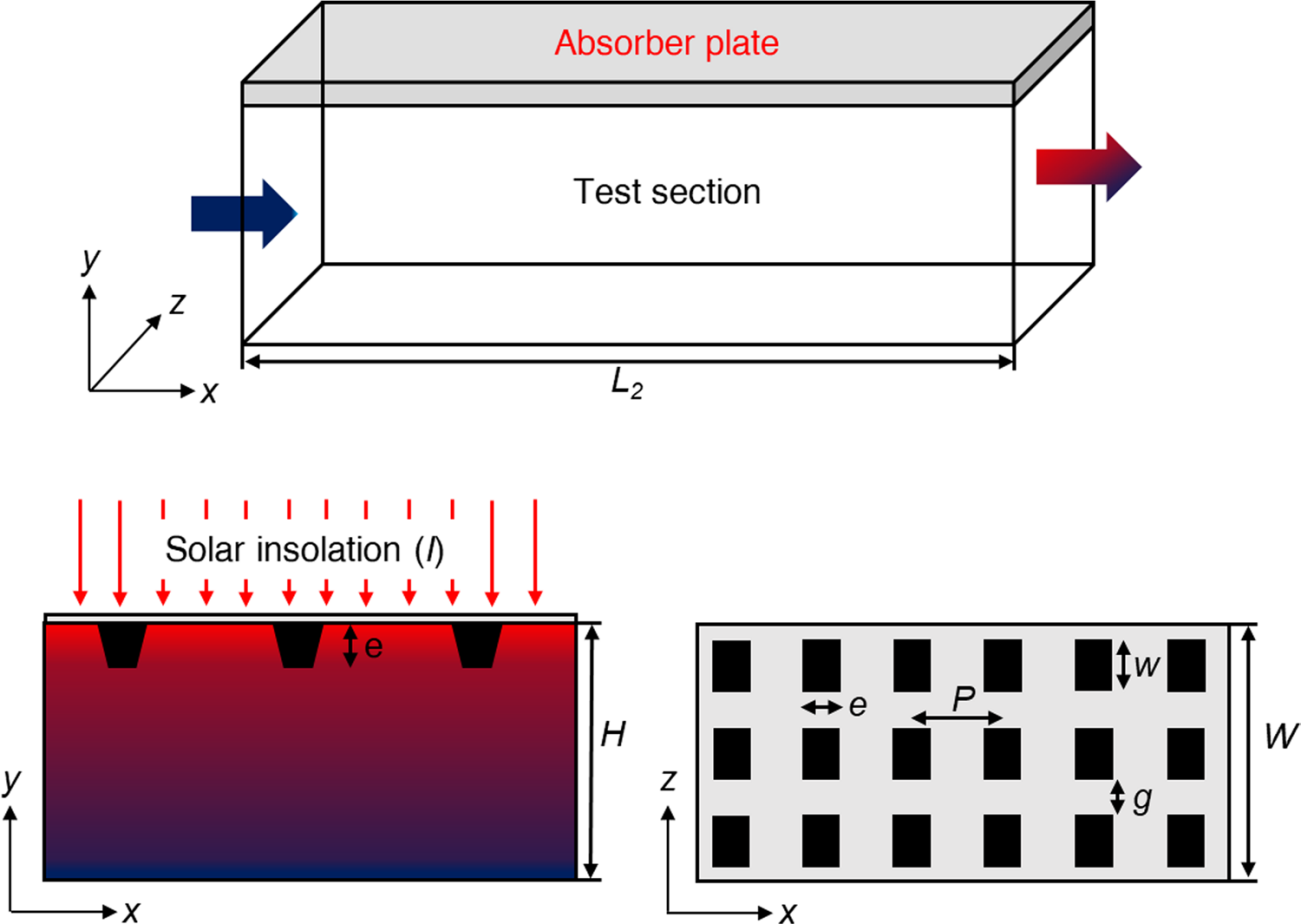
Schematic of a roughened SAH

**Fig. 2:**
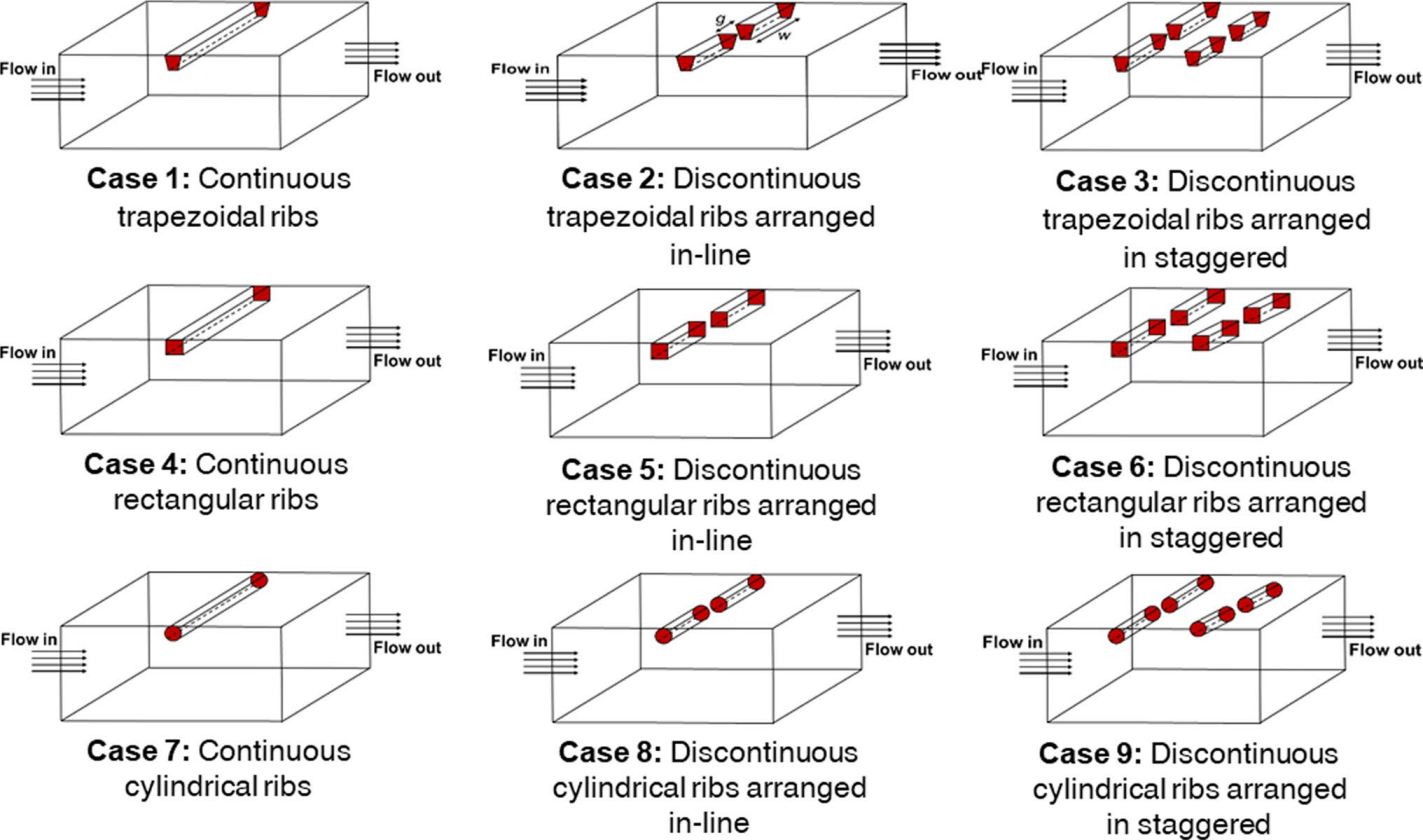
Schematics of the periodic computational domain used for investigation

The present investigated computational fluid domain is schematically illustrated in Fig. 1. The computational domain is created in the ICEM CFD package with artificial roughness attached on the underside surface of the absorber plate having different cross-sections: trapezoidal, circular, and square. The dimensions of the test section are considered to be 1000 mm × 300 mm × 25 mm. Table 2 contains the rib’s additional geometric details. It has been observed in the previous investigations (Tanda 2004b; Chaube et al. 2006) that the fluid flow becomes fully developed after interacting with the first three or four ribs. Thus, fluid flow behaves as a periodic flow (Singh et al. 2015, 2019b; Singh and Singh 2018). Based on this assessment, the periodic computational domain of one pitch length has been considered for further investigation. The artificial ribs are arranged in the transverse direction to the fluid flow in a rectangular roughened duct. Gaps are provided in the continuous transverse ribs, termed discontinuous ribs. Moreover, the transverse discontinuous ribs are attached to the absorber plate with two configurations, i.e., in-line and staggered. Three different types of cross-sectional ribs with two configurations along with continuous transverse ribs, making a total of 9 cases for the present investigation, are shown in Fig. 2.

**Table 2:** Roughened duct details used for the present CFD study

Parameters	Values
Test section length (*L*_2_)	1000 mm
Duct height (*H*)	25 mm
Aspect ratio (*W/H*)	12
Hydraulic diameter (*D*_*h*_)	46.154 mm
Rib height (*e*)	2 mm
Relative roughness height (*e/D*_*h*_)	0.043
Rib pitch (*P*)	16 mm
Relative roughness pitch (*P/e*)	8

### Physical modelling

**Fig. 3:**
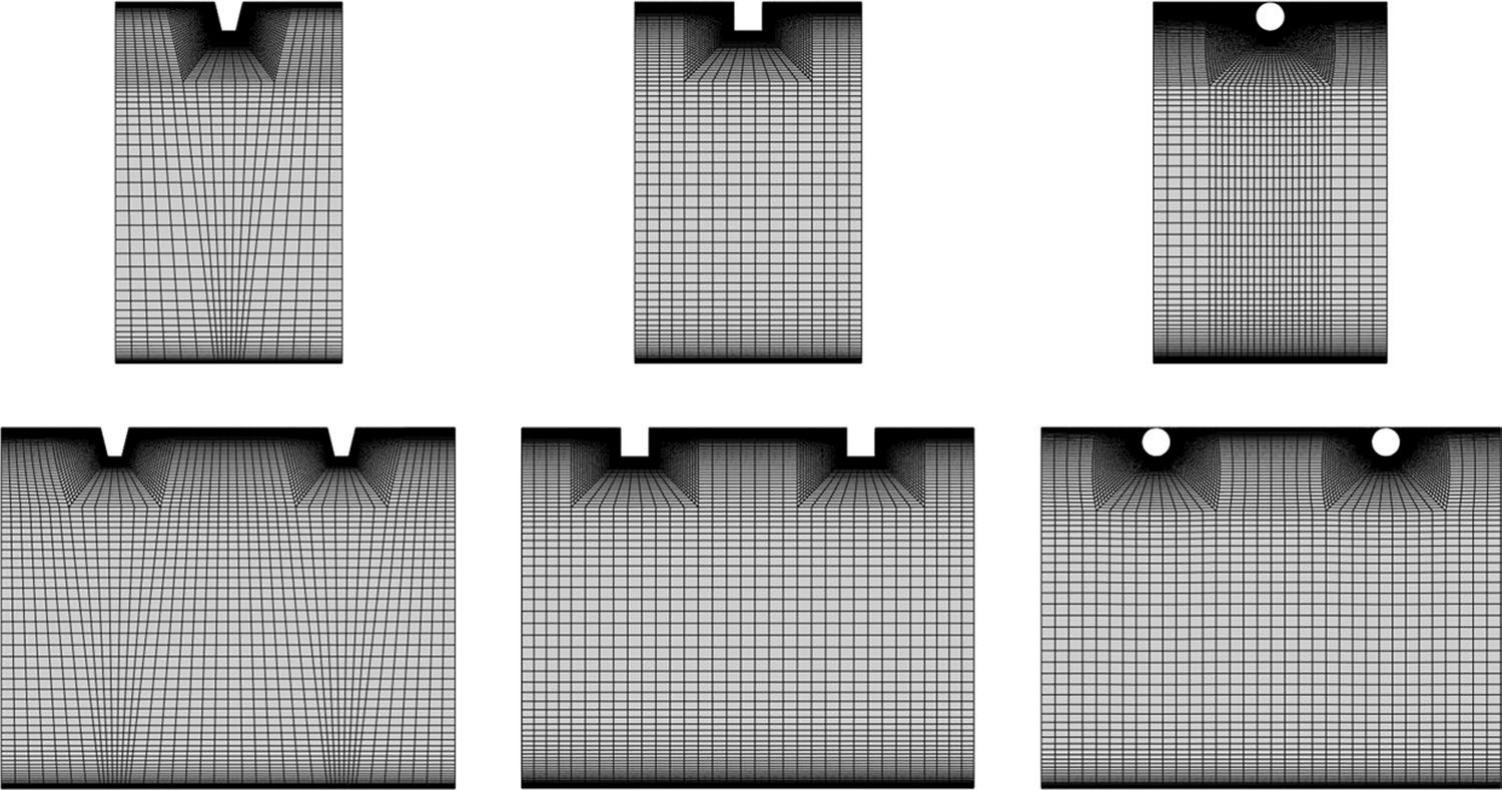
The discretization of the periodic computational domain for in-line and staggered configurations having different cross-sections

The periodic computational domain of roughened duct is discretized into structured non-uniform hexahedral volumes in 3-D planar in the ICEM CFD package to carry out the numerical simulations, as shown in Fig. 3. Some well-established assumptions have been considered for the present CFD analysis to reduce the complexity (Chaube et al. 2006; Singh et al. 2015; Singh and Singh 2018), which are stated below:

1. A steady fluid flow is assumed throughout the analysis.
2. An incompressible fluid is assumed throughout the domain.
3. The thermo-physical properties are considered constant throughout the analysis for both the fluid and the absorber plate.
4. Thin wall model for the absorber plate is considered (Singh et al. 2021).
5. The radiation is not considered in the analysis.

### Governing equations

Equations of conservation of mass (Eq. (1)), momentum (Eq. (2)), and energy (Eq. (3)) are solved in commercial software named ANSYS Fluent™ code 2021 R2 research license.

1 $$\nabla \bullet \left(\rho \bullet \overrightarrow{v}\right)=0$$

2 $$\nabla \bullet \left(\rho \bullet \overrightarrow{v}\bullet \overrightarrow{v}\right)=-\nabla p+\nabla \bullet \left(\mu \left[\left(\nabla \overrightarrow{v}+\nabla \overset{\acute{\mkern6mu}}{\overrightarrow{v}}\right)-\frac{2}{3}\nabla \bullet \overrightarrow{v}\ I\right]\right)$$

3 $$\nabla \bullet \left(\overrightarrow{v}\left(\rho E+p\right)\right)=\nabla \bullet \left({k}_{eff}\nabla T-h\overrightarrow{J}+\left(\mu \left[\left(\nabla \overrightarrow{v}+\nabla \overset{\acute{\mkern6mu}}{\overrightarrow{v}}\right)-\frac{2}{3}\nabla \bullet \overrightarrow{v}\ I\right].\overrightarrow{v}\right)\right)$$

### Boundary conditions

Required boundary conditions are applied on each wall of the considered computational domain. In addition, text user interface (TUI) commands are executed in the ANSYS fluent console window to redefine the default velocity inlet and outflow boundary conditions to periodic translational and periodic shadow conditions at the inlet and outlet, respectively. Text commands are used to define translational periodicity at the inlet by mesh/modify-zones/make-periodic/periodic zone-inlet/shadow zone-outlet/translationally periodic-yes/create periodic zones-yes. The inlet velocity boundary condition is changed to periodic where the constant mass flow rate is provided corresponding to the *Re*. Table 3 contains the range of operating parameters used in the CFD analysis. Constant heat flux is applied on the absorber plate of SAH, whereas walls other than the absorber plate are considered insulated. The outflow-type wall condition at the outlet is deleted permanently and defined as a periodic shadow. The thermo-physical properties used in the present CFD analysis are considered for *Pr* = 0.708 at *T* = 300 K (Singh et al. 2021). Moreover, the no-slip boundary conditions are made sure on the walls of the SAH.

**Table 3:** Operating parameters considered for the present CFD study

Parameters	Range
Mass flow rate ($\dot{m}$)	0.0151–0.0724 kg/s
Prandtl number (*Pr*)	0.708
Reynolds number (*Re*)	5000–24,000
Uniform heat flux (*I*)	1000 W/m^2^

### Numerical methodology

**Fig. 4:**
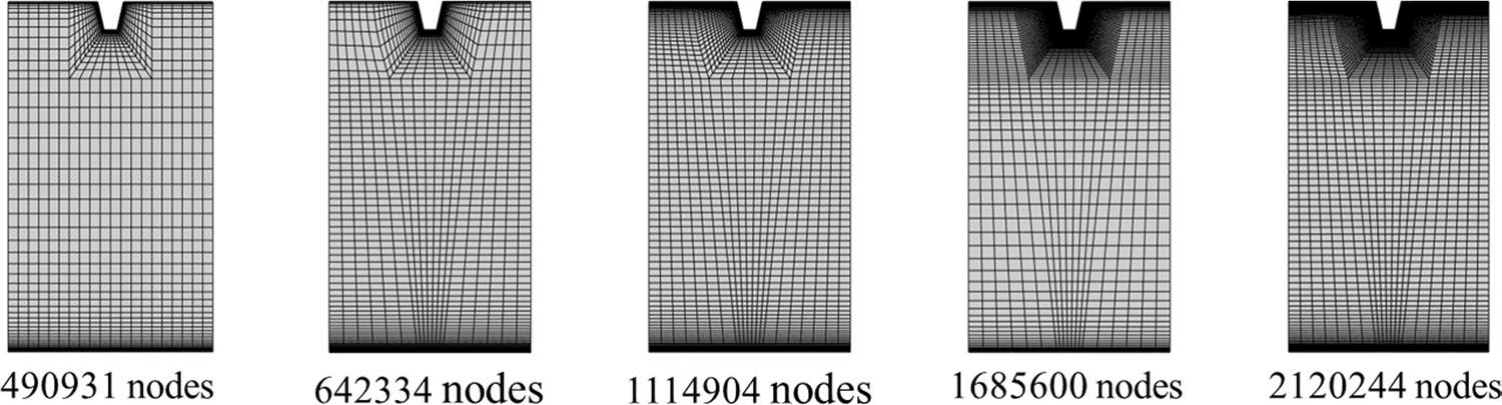
The discretized domain with a different number of nodes for the grid sensitivity test

**Fig. 5:**
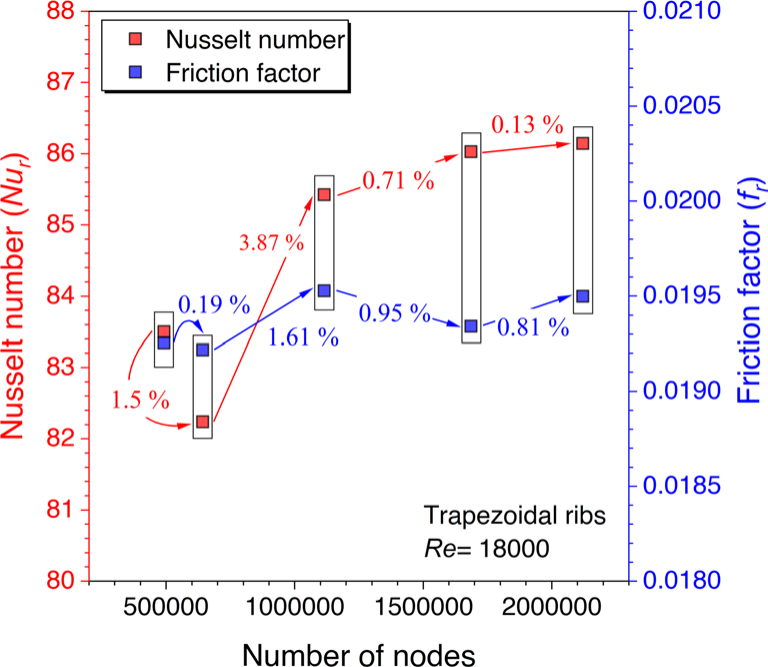
Grid sensitivity test

The double-precision pressure-based solver is used to solve the governing equations in three dimensions in a steady state for absolute velocity formulation. SIMPLE (semi-implicit method for pressure-linked equations) scheme is applied to couple pressure and velocity components. Least squares cell-based for gradient, a second order for pressure and a second-order upwind for momentum, turbulent kinetic energy, and dissipation rate is used for spatial discretization. The default relaxation factor is considered for the present investigation throughout the analysis. Residual convergence absolute criteria for mass, momentum, turbulence model terms, and energy are supposed to be of order 10^−6^, 10^−6^, 10^−6^, and 10^−8^, respectively. The computational domain of one pitch length having continuous transverse rib is discretized with structured non-uniform hexahedral volumes in the ICEM CFD package and ensured with fine meshes near the ribs to make sure a *y*^+^ value is equal to 1. In order to maintain *y*^+^ equal to 1, the first cell thickness was calculated with the help of the Reynolds number and the geometric parameters. To make sure that CFD results are grid-independent, discretization with the different numbers of nodes is done for grid sensitivity analysis, as illustrated in Fig. 4. CFD simulation has been conducted by the varying number of grid cells from 490,931 to 2,120,244 nodes. The percentage changes in the average Nusselt number and the friction factor have been found to be less than 1% after 1,114,904 nodes for two consecutive grid resolutions of the computational domain roughened by continuous trapezoidal ribs at Reynolds number 18,000 (see Fig. 5). Therefore, the grid size with 1,685,600 nodes is used for further analyses of the roughened duct, even with different cross-sectional ribs.

### Experimental validation for the smooth duct

Even though there are multiple theoretical correlations available in the literature for the smooth duct to calculate the heat transfer in terms of Nusselt number—Dittus-Boelter and Gnielinski correlations are the most widely used for validation purposes of the smooth ducts in solar-thermal applications. However, these correlations have their limitations, as discussed below:

- Dittus-Boelter equation (Taler 2013):

4 $$Nu=0.023\times {\mathit{\operatorname{Re}}}^{0.8}\times {\mathit{\Pr}}^{0.4},0.7\le \mathit{\Pr}\le 100;\mathit{\operatorname{Re}}\ge {10}^4;\frac{L}{D_h}\ge 60$$

It is clearly mentioned in the development of the Dittus-Boelter equation for cylindrical ducts that its validity works over *Re* = 10,000. In addition, the length of the test section should be more than sixty times the hydraulic diameter. It means most investigated SAH ducts do not fall into this category. However, the Gnielinski correlation gives more promise for smooth duct heat transfer validation because its validity ranges from a low regime of Reynolds number to a higher regime without any restrictions for the test section length.

Gnielinski correlation (Taler 2013):

5 $${\displaystyle \begin{array}{c} Nu=\frac{\left[\left(\frac{f}{8}\right)\left(\mathit{\operatorname{Re}}-1000\right)\mathit{\Pr}\right]}{\left[1+12.7\surd \left(\frac{f}{8}\right)\left({\mathit{\Pr}}^{\frac{2}{3}}-1\right)\right]}\\ {}f={\left[0.79\ln \left(\mathit{\operatorname{Re}}\right)-1.64\right]}^{-2},2300<\mathit{\operatorname{Re}}<{10}^6;0.5<\mathit{\Pr}<2000\end{array}}$$

Therefore, an experimental study is carried out for the smooth duct validation for more clarification regarding theoretical correlations, and the details of the experimental study are provided in the further subsections.

#### Experimental setup

**Fig. 6:**
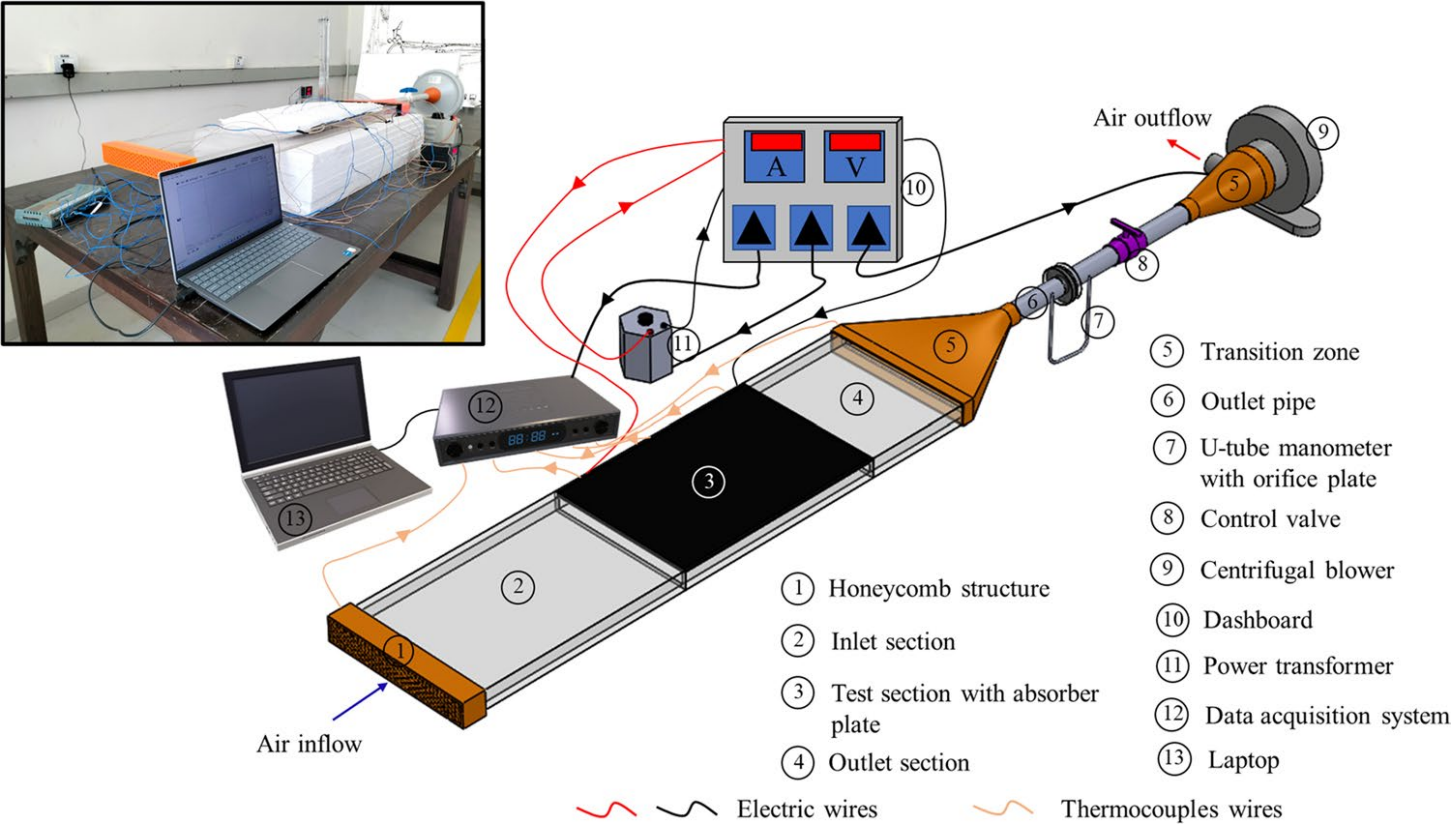
The pictorial and schematic view of the experimental setup

An experimental test setup is manufactured into a rectangular duct for smooth duct validation. It contains three main sections: inlet, outlet, and test. The inlet section is kept 500 mm long, while the outlet section is 250 mm long as per ASHRAE standard (Standards A, November C, Board A, Janu-D, 1991), more than 5√*W* × *H* and 2.5√*W* × *H*, respectively. The test section length is kept 450 mm long, where the aluminum sheet of 6 mm thickness is atop the test section. The pictorial view of the experimental test rig is provided in Fig. 6. The schematic is provided to identify the individual components used in the experimental test setup. The walls of the experimental test rig are made up of acrylic glass, which has low thermal conductivity and thus reduces the heat transfer losses to the surrounding. The honeycomb structure at the inlet supports the inlet section to induct the air uniformly in the duct. This structure has hexagonal meshing, which is 3-D printed—polylactic acid (PLA) material is used. The flat plate mica sheet heaters are used to heat the absorber plate, and the top of the heaters are fully insulated with an aluminum silicate high-temperature insulation ceramic fiber blanket. The input supply is controlled with a variable power transformer, which has a capacity of 2 A, and output voltage may vary from 0 to 270 V. Therefore, the voltmeter and ammeter are used to indicate the input power supply’s voltage and current, respectively. The rectangular outlet section is attached to the centrifugal blower of 0.5 HP capacity with the help of transition zones, which are also 3-D printed to perfectly match the two distinct cross-sections. These connecting points have the orifice meter with a U-tube manometer and the control valve. The control valve maintains the required flow rate in the duct at the inlet. Calibrated K-type copper-constantan thermocouples are attached at different locations to monitor the temperature reading in the experimental test rig. A total of six thermocouples are connected at the inlet and outlet of the rectangular duct—three each on the inlet and outlet. In addition, the ten thermocouples are attached to the absorber plate surface to measure the wall temperature. These thermocouples record the temperature reading and transfer to the computer connected with the help of the data acquisition system. The experimental test rig is mounted on the foundation provided with the thermocol sheets below the test section—which helps maintain equal levels between the rectangular duct’s inlet and the inlet of the centrifugal blower, thus reducing the heat transfer losses through the bottom surface of the duct to the surrounding.

#### Experimental procedure

Before recording the readings from the experimental test rig, each component is checked, and the test is run to achieve a steady flow rate. It took around 2.5 h to achieve steady state for each run. A total of six runs were performed to get the data for the required Reynolds number, and each run was performed at least twice to check repeatability. The following data are extracted for each run:

•The mass flow rate at the inlet to get the required Reynolds number is calculated as:

6 $$\dot{m}=\rho {A}_cV$$

•The temperature readings at the outlet and inlet.

A total of six thermocouples are fixed at the inlet and outlet. Therefore, the arithmetic mean of three thermocouples is used to calculate the average *T*_*i*_ and *T*_*o*_.

7 $${T}_i=\frac{\left({T}_1+{T}_2+{T}_3\right)}{3},{T}_o=\frac{\left({T}_4+{T}_5+{T}_6\right)}{3}$$

•The temperature reading at the absorber plate.

A total of ten thermocouples are used to record the absorber plate temperature, and the average wall temperature is the arithmetic mean of all ten thermocouples.

8 $${T}_w=\frac{\left({T}_7+{T}_8+{T}_9+{T}_{10}+{T}_{11}+{T}_{12}+{T}_{13}+{T}_{14}+{T}_{15}+{T}_{16}\right)}{10}$$

**Fig. 7:**
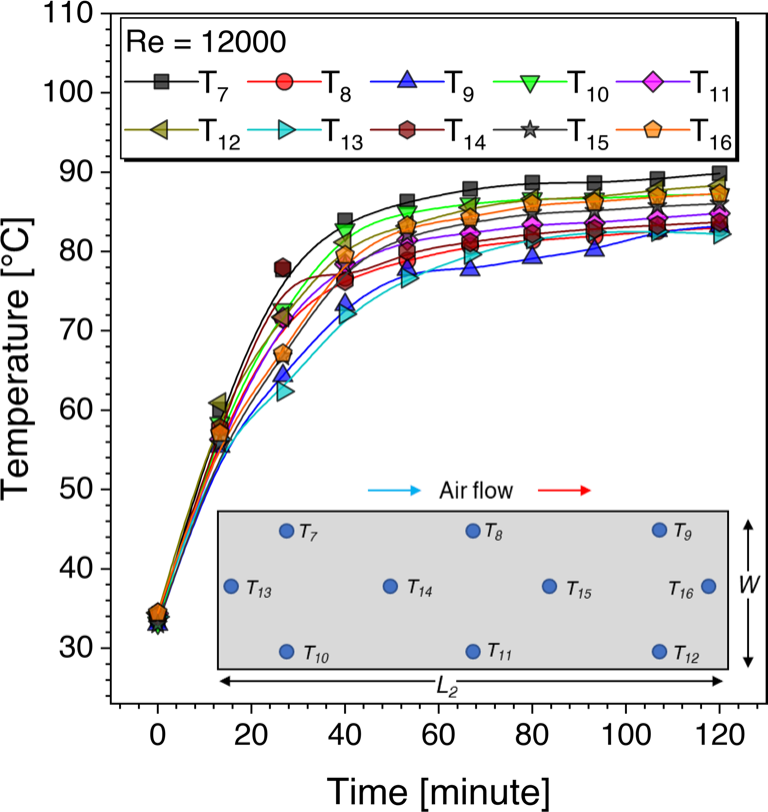
Absorber plate temperature distribution for *Re* = 12000

Figure 7 gives spatial distribution of temperature recorded for smooth absorber plate at Reynolds number of 12000. It may be seen a steady state is achieved after 2 h. These readings are used to calculate the average convective heat transfer coefficient, and further, the Nusselt number values are computed for each Reynolds number value.

#### Experimental validation

**Fig. 8:**
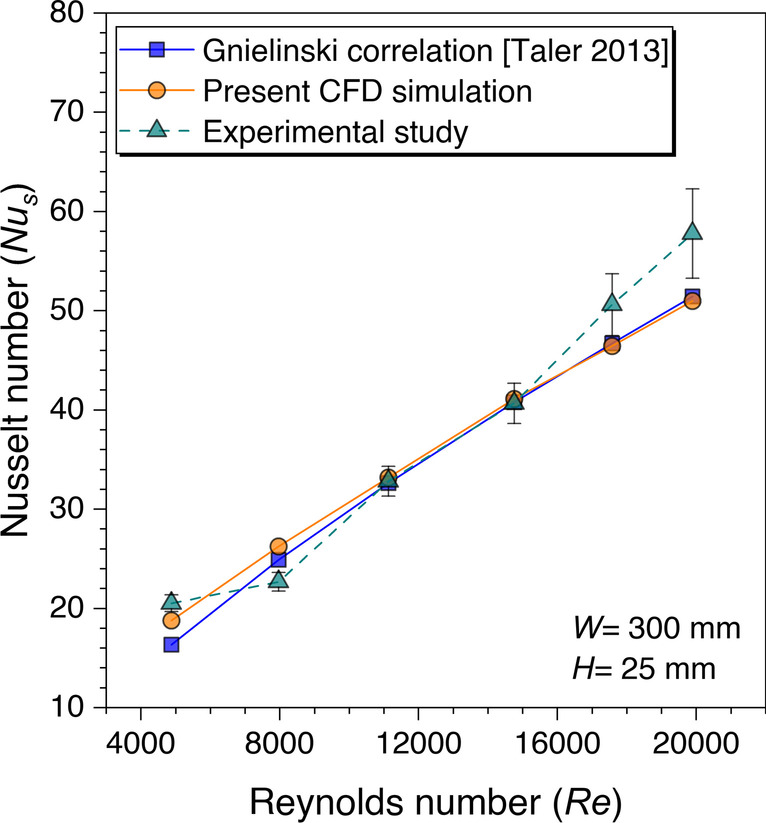
*Nu*_*s*_ values validation against *Re* for smooth duct

From Eqs. (4) and (5), it is concluded that the Dittus-Boelter equation is valid for higher Reynolds numbers and higher length-to-hydraulic diameter ratio, however, the Gnielinski correlation is perfectly suitable for the SAH operational conditions. Therefore, it is concluded that the Gnielinski is more accurate for the heat transfer calculation in the smooth duct (Winterton 1998). Hence, an experimental study is carried out for smooth duct validation keeping the same geometric conditions taken for CFD investigation. The experimental values are plotted against the CFD simulation values, as shown in Fig. 8. The experimental values are plotted with the uncertainty error bar calculated for each Reynolds number. The details of the uncertainty range associated with each value are provided in Appendix A. The same trend is observed in the experimental values as in the CFD simulation values that on increasing the Reynolds number values, the Nusselt number values are increasing.

In addition to the Nusselt number validation, the CFD friction factor values are also validated with the theoretical correlation. Similarly, there are multiple theoretical correlations available in the literature for the friction factor. However, the Blasius correlation and Bhatti and Shah correlation are found to be very popular for the SAH validation. Though the effectiveness of both correlations is observed to be satisfactory but the Bhatti and Shah correlation does consider the aspect ratio of the annular duct, therefore, this correlation is more suitable for the SAH duct. Moreover, the Blasius equation is developed on the data collected for the circular section duct. The details of both correlations are given below (Karwa et al. 1999):

Bhatti and Shah correlation (Karwa et al. 1999):

9 $${\displaystyle \begin{array}{c}f=\left(1.0875-0.1125\frac{H}{W}\right){f}_c\\ {}{f}_c=0.0054+2.3\times {10}^{-8}{\mathit{\operatorname{Re}}}^{1.5};2300<\mathit{\operatorname{Re}}<4000\\ {}=1.28\times {10}^{-3}+0.1143{\mathit{\operatorname{Re}}}^{-0.311};4000<\mathit{\operatorname{Re}}<{10}^7\end{array}}$$

Blasius equation (Taler 2013):

10 $$f=0.0791{\mathit{\operatorname{Re}}}^{-0.25}$$

Modified Blasius equation (Taler 2013):

11 $$f=0.085{\mathit{\operatorname{Re}}}^{-0.25}$$

**Fig. 9:**
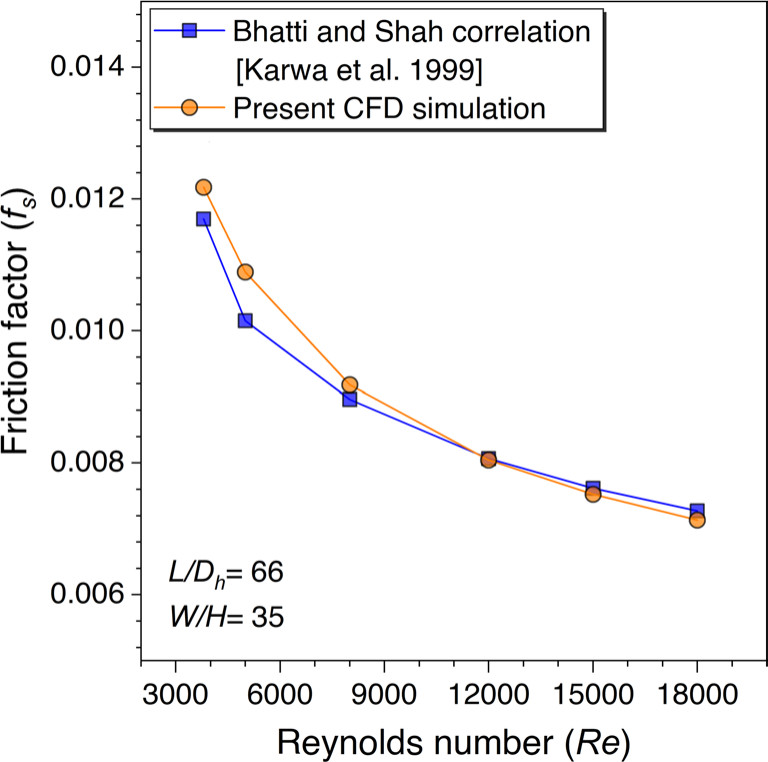
*f*_*s*_ values validation against *Re* for smooth duct

Therefore, the numerical frictional factor values are plotted against the Bhatti and Shah correlation values for *Re* ranging from 3800 to 18,000. The comparative results are presented in Fig. 9. The CFD values are observed to be conformal to the theoretical correlation values; hence, it is concluded that the Bhatti and Shah correlation is the best fit for the friction factor values for smooth duct validation.

### Roughened duct validation

**Fig. 10:**
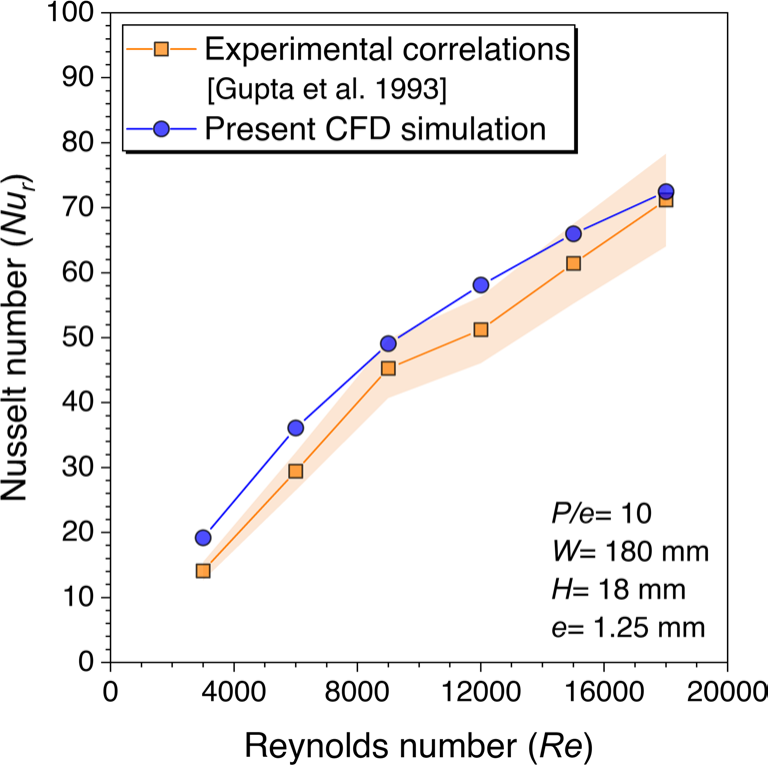
*Nu*_*r*_ values validation against *Re* for roughened duct

**Fig. 11:**
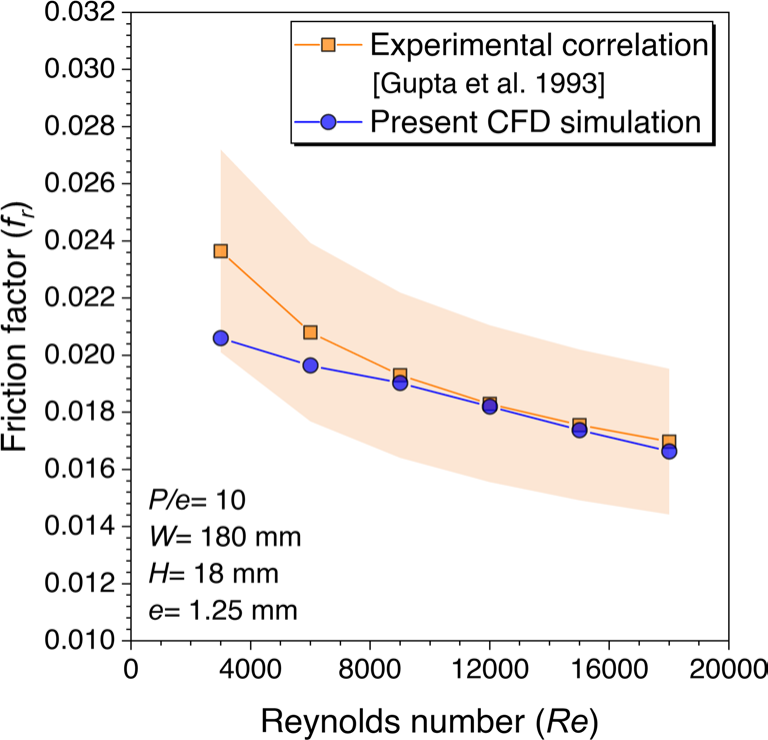
*f*_*r*_ values validation against *Re* for roughened duct

Correlations developed by Gupta et al. (1993) for *Nu*_*r*_ and *f*_*r*_ based on experimental data have been used to perform the validation for roughened duct. Rectangular duct height of 18 mm roughened with fixed transverse cylindrical rib diameter of 1.25 mm having *P/e* = 10 is considered for validation with one pitch length. The numerical values of *Nu*_*r*_ are observed to be very close to the correlation values plotted with the ±10% error band, as shown in Fig. 10. The renormalization-group (RNG) *k*-*ε* turbulence model with enhanced wall treatment was used to analyze the thermal-fluid behavior roughened with static cylindrical ribs in the SAH duct. The absolute mean percentage deviation of the *Nu*_*r*_ values was observed to be in the range given in the paper with the correlation values. The numerical values of *f*_*r*_ are observed to be very close to the correlation values plotted with ±15% error band except at *Re* = 3000 because this range comes under the transition zone of internal flow, as shown in Fig. 11. This was the basis for selecting the RNG *k*-*ε* with enhanced wall treatment turbulence model for studying the heat transfer and fluid flow behavior with transverse continuous and discontinuous ribs having different cross-sections in a roughened SAH.

## Thermal enhancement factor calculation

The thermal enhancement factor in terms of THPF is calculated with the help of the *Nu* and *f* ratio with respect to the smooth duct values. This factor is developed for equal pumping power in smooth and roughened ducts. It means if the value is reported more than one—depicts the better performance than the smooth duct. The procedure to calculate the thermal enhancement factor is discussed below:

Total heat absorber by the air in a rectangular duct is measured by:

12 $${Q}_u=\dot{m}{c}_p\left({T}_o-{T}_i\right)$$

The incoming mass flow rate at the inlet is calculated by:

13 $$\dot{m}=\rho {A}_cV$$

The total heat transfer to the fluid by convection is measured by:

14 $${Q}_c=h{A}_s\left({T}_w-{T}_m\right)$$

As per the assumption considered during the analysis:

15 $${Q}_u={Q}_c$$

The average convective heat transfer coefficient is defined by:

16 $${h}_{avg}=\frac{1}{A}\int hdA$$

The average Nusselt number is calculated by:

17 $${Nu}_{avg}=\frac{h_{avg}\times {D}_h}{k_{air}}$$

The rectangular diameter is defined as:

18 $${D}_h=\frac{4{A}_c}{P_{wetted}}$$

The friction factor is defined as:

19 $${f}_r=\frac{\left(\frac{\Delta P}{l}\right)\times {D}_h}{2\rho {v}^2}$$

Thermo-hydraulic performance factor (THPF) (Webb and Eckert 1972) is defined by:

20 $$THPF=\frac{\left({Nu}_r/{Nu}_s\right)}{{\left({f}_r/{f}_s\right)}^{1/3}}$$

## Results and discussion

CFD simulations of transverse discontinuous ribs with three different cross-sections arranged in two configurations, viz., in-line and staggered, have been performed to investigate the thermal-fluid behavior of roughened SAH with discontinuous ribs. The effects of different design parameters are taken into consideration. Additionally, *Nu* for heat transfer and *f* for pressure drop have been calculated in each case for optimization of SAH with discontinuous ribs. Moreover, the rectangular and cylindrical ribs are also simulated for the thermal performance comparison with the trapezoidal ribs. Furthermore, the analysis of fluid behavior and its impact on thermal performance are also discussed.

### Effects of gap width (*g*) in transverse discontinuous ribs

**Fig. 12:**
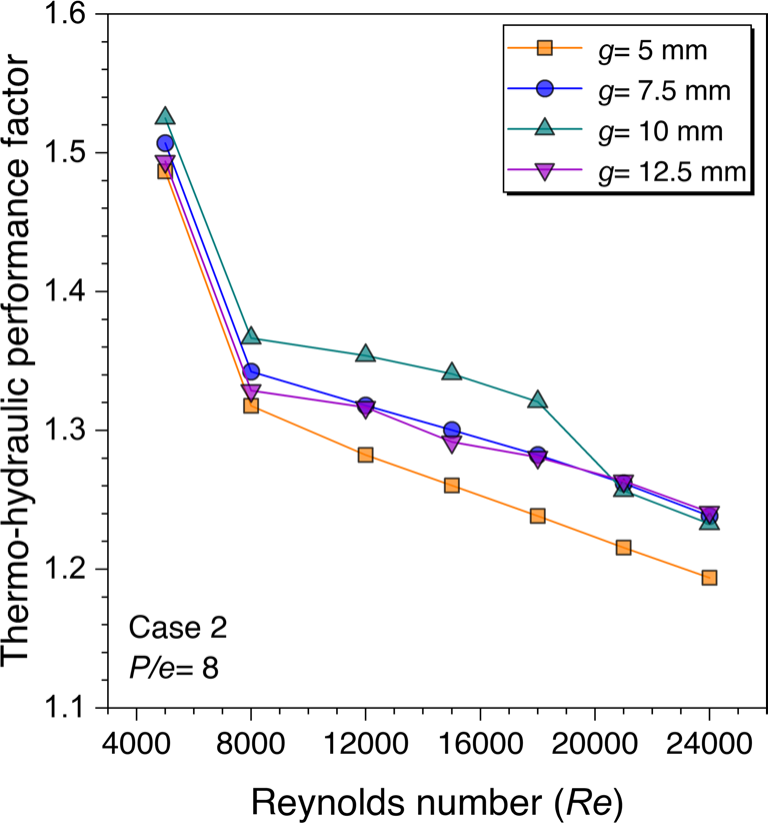
Variation of THPF against *Re* for gap width optimization

Since this is found in the literature survey that the placement of the ribs and their shapes play a significant role in augmenting the heat transfer, therefore, to understand the effects of the gap provided in transverse discontinuous ribs on thermal-fluid characteristics, four different values are considered for the analysis. The gap values are reported in terms of gap width and denoted by the letter “*g*.” The gap width values are provided in transverse ribs having trapezoidal cross-sections for parametric optimization of the gap width. The gap width values ranging from 5 to 12.5 mm are considered for numerical analysis. The rib height (*e*) is considered constant and equal to 2 mm whereas the corresponding relative roughness pitch is considered *P/e* = 8. The variation in THPF values is presented in Fig. 12 against the *Re* for different values of rib gap width.

It is interpreted from Fig. 12 that the THPF values decrease on increasing the *Re* values. However, as the gap width value increases, the THPF values are found to be increasing until *g* = 10 mm, after that the THPF values are observed to be lower. It means as the rib gap width is increasing, the high stream fluid that comes out of the gaps has attained a maximum with *g* = 10 mm, and it starts decreasing after this value. Moreover, *g* = 10 mm rib gap width outperformed the other considered rib gap width values before *Re* = 21,000; however, the values are approximately the same or below at *Re* = 21,000 and 24,000. Hence, it is concluded that *g* = 10 mm is the optimum value for maximum THPF in the present study.

Since THPF is a dimensionless factor that accounts for the combined effects of the Nusselt number and friction factor change in a roughened duct over a smooth duct, therefore, its value is highly dependent on the Nusselt number and friction factor ratio (see Eq. (20)). Initially, at the lower Reynolds number, the Nusselt number ratio is very high with respect to the friction factor ratio, thus its effect is visible in the high value of THPF, however, as the Reynolds number value increases the change in the Nusselt number ratio over the friction factor ratio is lower, hence the THPF values are decreased sharply on increasing the Reynold number values and becomes flatter.

**Fig. 13:**
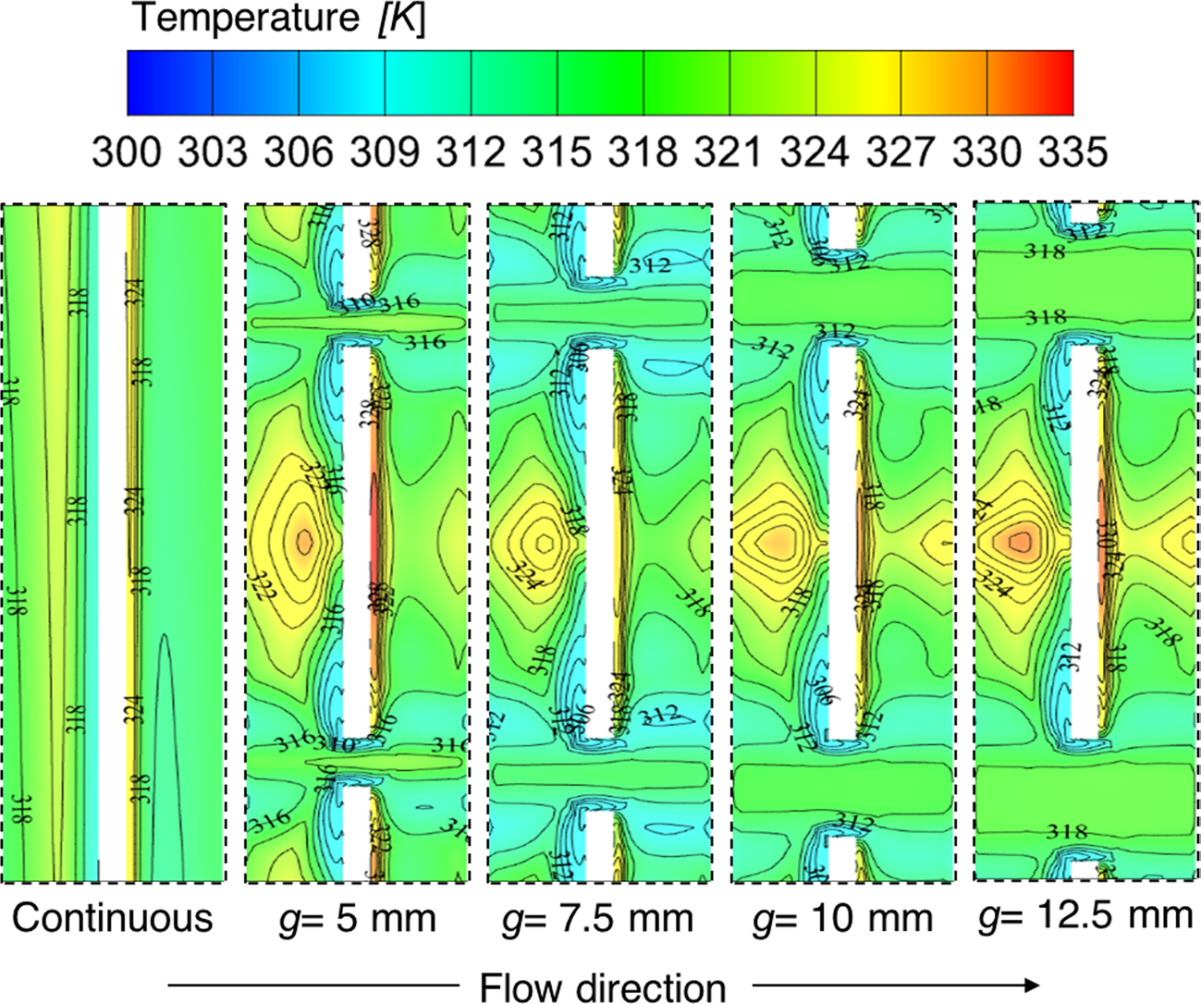
Temperature distribution on absorber plate for different gap widths along with continuous transverse rib

To attest to the reasoning behind the optimum value of *g* = 10 mm, the temperature variation on the absorber plate is illustrated in Fig. 13. The temperature contours are extracted from the numerical analysis for different gap width values and compared with the continuous transverse rib. It is observed from the temperature contours that the temperature value is higher in downstream regions because the fluid particles stick behind the continuous transverse ribs and form a re-circulation zone. However, the gaps in the discontinuous ribs allow the stuck fluid particles to flow through the gaps. It is also understood from the temperature contours with different gap widths that the hotspot region formed behind the continuous transverse ribs starts getting dispersed. The high-energy fluid particles get the space to mix with the low-energy fluid particles as lower temperature values are found between the ribs. As the gap width increases, the small hotspots formed behind discontinuous ribs also start diffusing. The diffusion attained its maximum at *g* = 10 mm. After that, the hotspot formation starts again because the fluid particles are quickly getting out through the gaps; this is observed from the temperature contours with lines shown in Fig. 13. Hence, with the help of temperature contours, it is attested that the value *g* = 10 mm is the optimum value for further analyses.

**Fig. 14:**
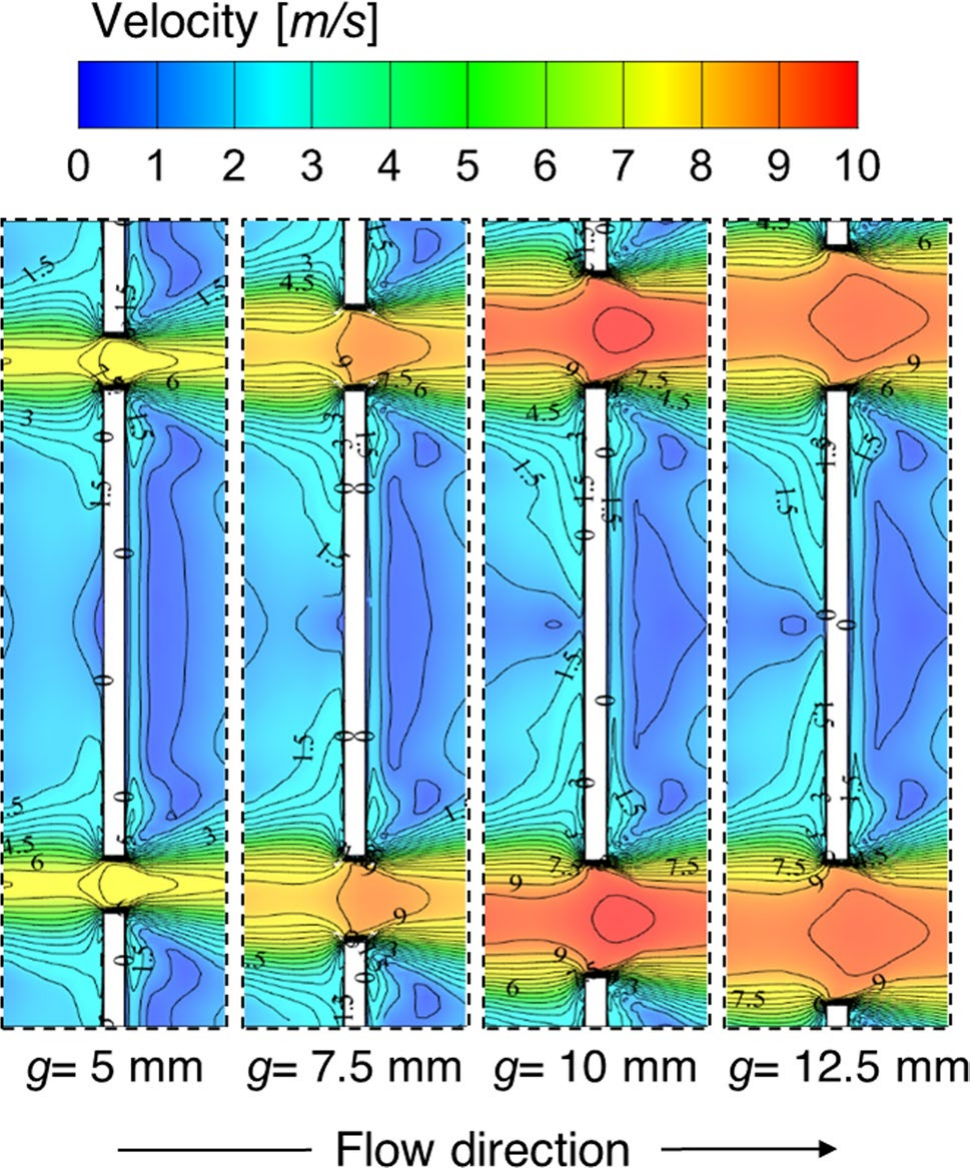
Velocity contour with lines at 1 mm below parallel to absorber plate for different gap widths

Further, to understand the reason supporting this optimized gap width size, the velocity contours are provided in Fig. 14. The velocity contours are extracted from the simulated cases for different values of gap width at 1 mm below an absorber plate. It is seen from the contours that the fluid was supposed to be stuck behind the continuous transverse ribs in the downstream region and can now pass through the gaps provided in the discontinuous ribs. As the gap size increases, the stuck fluid particles start passing through the path formed between the two consecutive discontinuous ribs. The diffusion of the attached fluid particles starts happening on both sides of the discontinuous rib—this diffusion results in more mixing of high-energy fluid particles with less-energy particles. The distribution of attached fluid particles is easily observed in velocity contours plotted with lines. The dying nature of settled fluid particles is started with the lower value of gap width-sized and fully attained maximum at *g* = 10 mm. This also contributed to the high streams of fluid particles through the gap. After this gap size, again, the formation of re-circulation of fluid particles starts as the large gap size allows fluid particles to flow easily without disturbing the nearby fluid zone. Hence, this is concluded from the velocity contours that *g* = 10 mm is the optimum value amongst all the considered values of gap width.

### Effects of the discontinuous rib width (*w*)

**Fig. 15:**
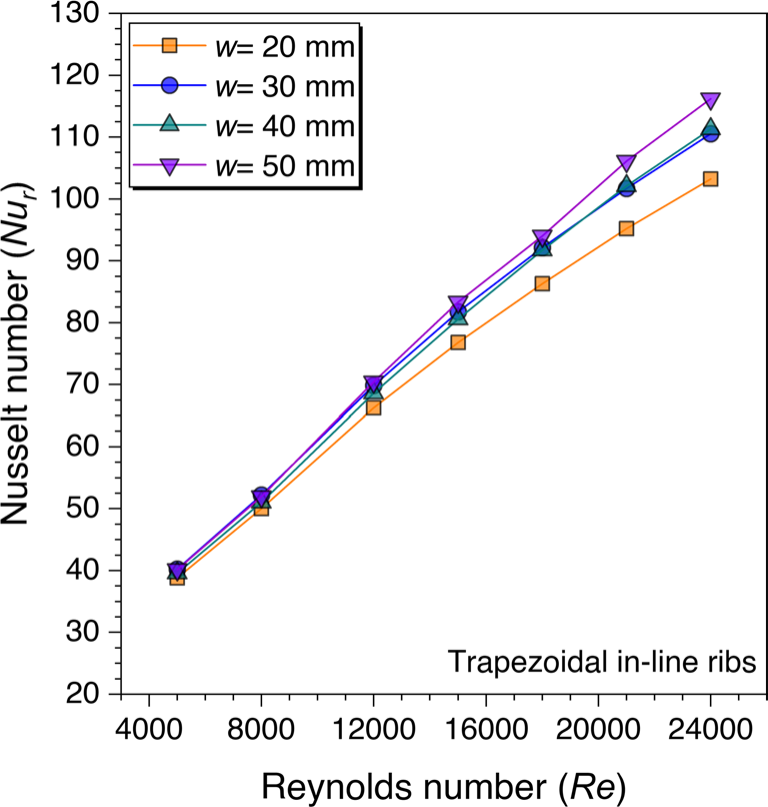
Variation of *Nu*_*r*_ against *Re* for discontinuous rib width optimization

Since the gap width size in the discontinuous ribs is optimized in the previous section, therefore, the different values of discontinuous rib width are considered in this section for geometric optimization. The discontinuous rib width is denoted by the letter “*w*,” and the effects of different values are plotted in Fig. 15. Four values of discontinuous rib width are considered from 20 to 50 mm. It is observed from Fig. 15 that the *Nu*_*r*_ values are increasing on increasing the *Re* values. This happened because the heat transfer area is increased with an increase in the discontinuous rib width value as fewer gaps are present on the absorber plate. However, the *Nu*_*r*_ values are observed to be the same with *w* = 30 mm and 50 mm until *Re* = 12,000, after that the discontinuous rib width size equal to 50 mm outperforms the other considered values in terms of maximum *Nu*_*r*_ in the present study. This phenomenon happened because the heat transfer surface area is almost the same with *w* = 30 mm and 50 mm; however, this effect diminished after *Re* = 12,000. Hence, it is concluded that *w* = 50 mm is the optimum discontinuous rib width size in terms of maximum *Nu*_*r*_ in the present study.

**Fig. 16:**
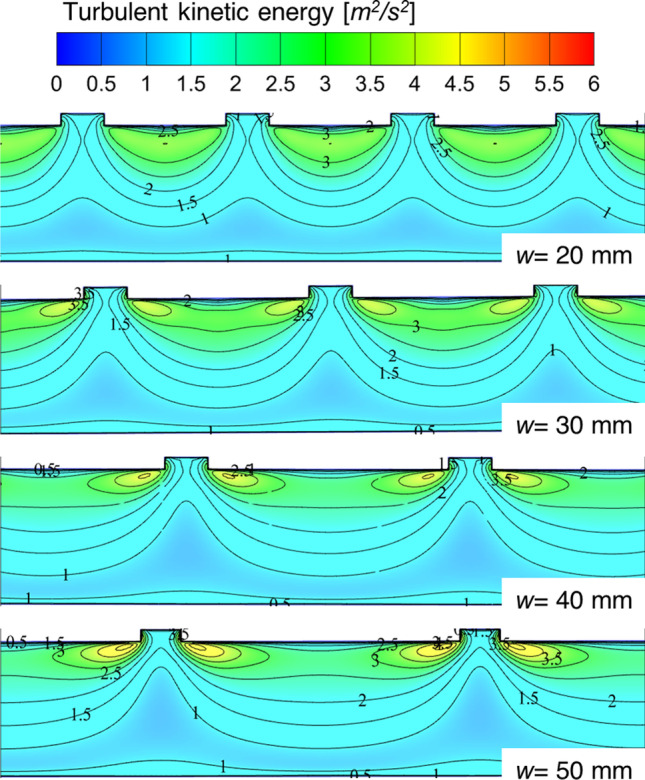
Turbulent kinetic energy contours for different values of discontinuous rib width

The other cause of enhancing the *Nu*_*r*_ values is observed in the turbulent kinetic energy contours, illustrated in Fig. 16*.* The turbulent kinetic energy values are increasing continuously with an increase in the discontinuous rib width size. The maximum turbulent kinetic energy (TKE) value increased from 3 to 6.15 m^2^/s^2^ for *w* = 20 mm to 50 mm, respectively. These values are directly associated with enhanced heat transfer rate in the fluid domain. The highest peaks of the TKE are found to be near the sharp corners of the discontinuous rib.

**Fig. 17:**
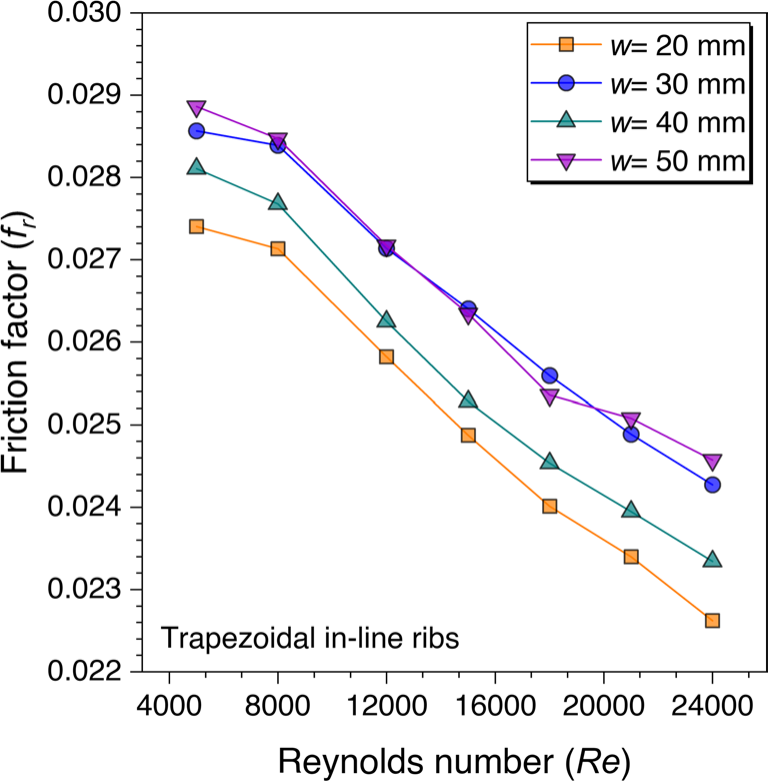
Variation of *f*_*r*_ against *Re* for gap width optimization

Figure 17 shows the variation of the *f*_*r*_ value against the *Re* for different values of the discontinuous rib width. It is observed that the *f*_*r*_ values are decreasing continuously on increasing the Reynolds number values. This is because of subduing of the end wall effect associated with discontinuous ribs’ sharp edges. The pressure drops across the test section, which results in *f*_*r*_ values, is observed less with *w* = 20 mm as more gap is available to flow the fluid freely. But the friction factor values resulted differently for other values of discontinuous rib width. The friction factor values are observed to be approximately equal in numerical values with *w* = 30 mm and *w* = 50 mm. The reason is attributed to the end wall effect and discontinuous rib width. As the discontinuous rib width increases from *w* = 20 mm to *w* = 30 mm, the number of discontinuous ribs decreases, resulting in less free space for fluid flow. That is why the friction factor values for *w* = 30 mm increased. Additionally, the end wall effects also contributed to enhancing the *f*_*r*_ values. Similarly, the case is approximately the same with *w* = 50 mm. However, the friction factor values are observed to be less for *w* = 40 mm than *w* = 30 mm and 50 mm, but higher than the *w* = 20 mm.

**Fig. 18:**
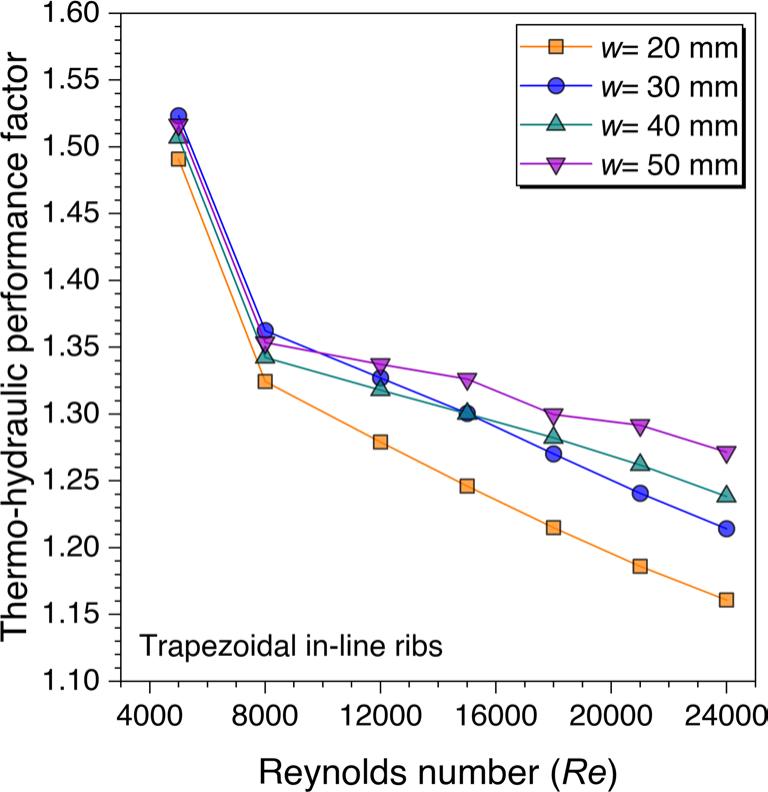
Variation of THPF against *Re* for gap width optimization

Since it needs to be clarified by observing the combined effects of the Nusselt number and friction factor values variation against the Reynolds number to get the optimum value of *w*, the variation of the THPF value against the Reynolds number is plotted in Fig. 18 as this factor takes care of both parameters simultaneously. The THPF values are observed to decrease on increasing the Reynolds number values for all considered values of discontinuous width. However, the maximum THPF value is first yielded for *w* = 30 mm till *Re* = 10,000, after that the discontinuous rib width equal to 50 mm outperforms the other considered values in the present study. This trend reflects the cumulative effect of the Nusselt number and friction factor values as the Nusselt number was also found maximum after *Re* = 8000 whereas friction factor values were approximately the same or lower after *Re* = 12,000 than *w* = 30 mm. Hence, it is concluded from Fig. 18 that the optimum value is observed to be *w* = 50 mm for all Reynolds number values in the present study.

### Comparison of discontinuous ribs over continuous ribs having similar cross-sections

**Fig. 19:**
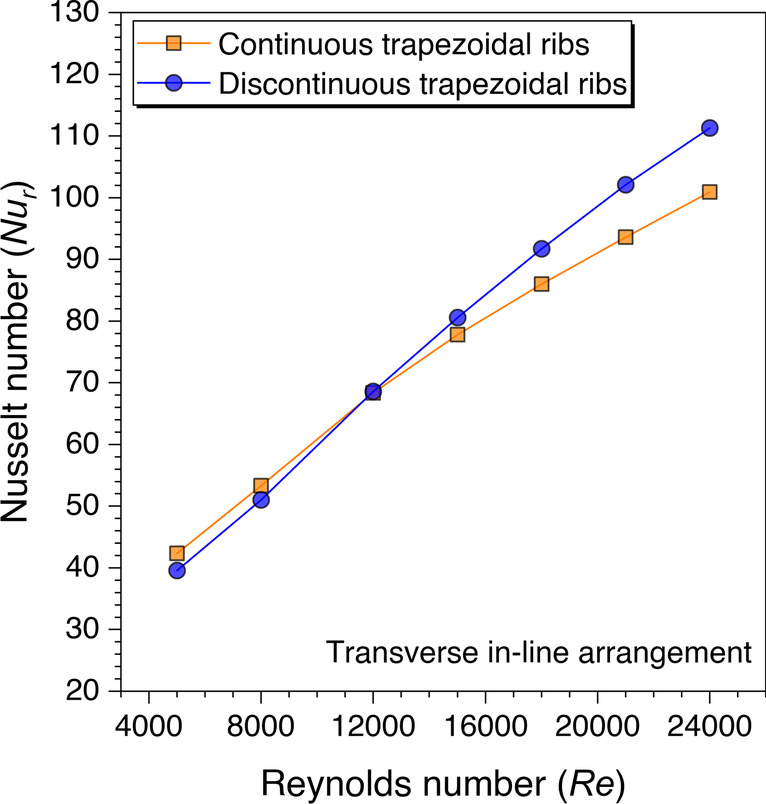
Variation of *Nu*_*r*_ against *Re* for trapezoidal ribs

**Fig. 20:**
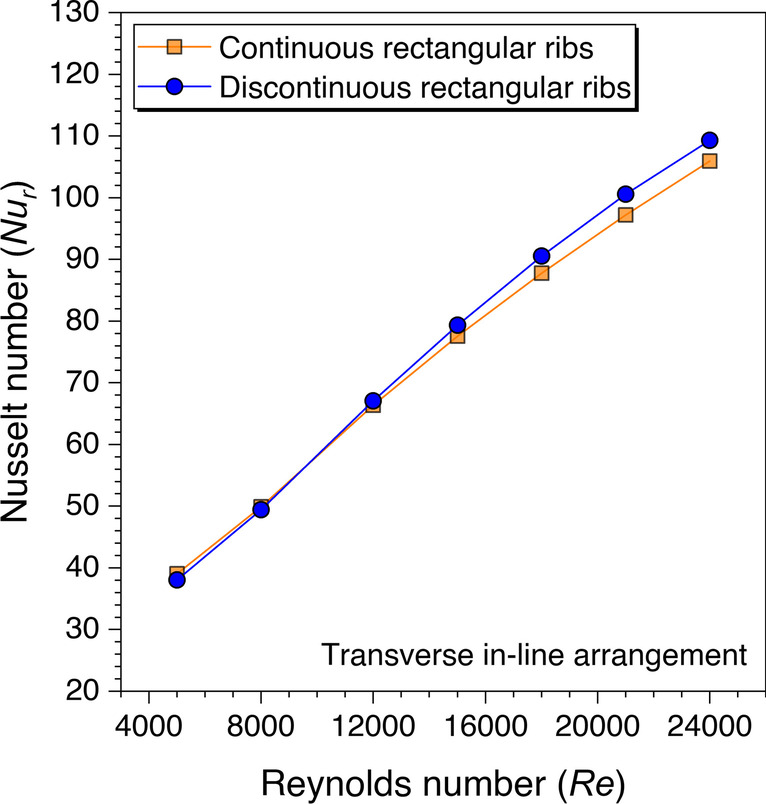
Variation of *Nu*_*r*_ against *Re* for rectangular ribs

Since the parametric optimization of discontinuous ribs having trapezoidal ribs is completed in the previous sections, the same values are taken for further analysis of discontinuous ribs having square and circular cross-sections. Figure 19 shows the *Nu*_*r*_ values variation against the *Re* for trapezoidal ribs. The *Nu*_*r*_ values are plotted to compare the discontinuous ribs over continuous ribs. It is observed that the discontinuous ribs performed better than the continuous ribs after *Re* = 12,000. This happened because the high stream fluid particles pass through the gaps in the discontinuous ribs, leading to a high local heat transfer coefficient resulting in heat transfer augmentation. Though the *Nu*_*r*_ values are found better with the continuous ribs until *Re* = 12,000, because of the dominant factor of the heat transfer area, however, this factor minimizes after *Re* = 12,000, and the heat transfer augmentation is dominated by the high stream fluid particles passing through the gaps in the discontinuous ribs. Hence, it is concluded that the discontinuous ribs perform better with the higher Reynolds number range than trapezoidal ribs in the present study. Moreover, the Nusselt number variation against the Reynolds number is also studied for the rectangular ribs to gain an understanding of the heat transfer; therefore, the results are plotted in Fig. 20.

A similar trend is observed with the rectangular ribs as with the trapezoidal ribs that the *Nu*_*r*_ values are found better with the discontinuous ribs over continuous ribs after *Re* = 12,000, as shown in Fig. 20. The same physics is also involved in the heat transfer augmentation with the rectangular ribs but the maximum Nusselt number values are obtained with the trapezoidal ribs. This is because the fluid particles are more attached to the trapezoidal ribs in comparison to the rectangular ribs as there are fewer sharp edges with the trapezoidal ribs.

**Fig. 21:**
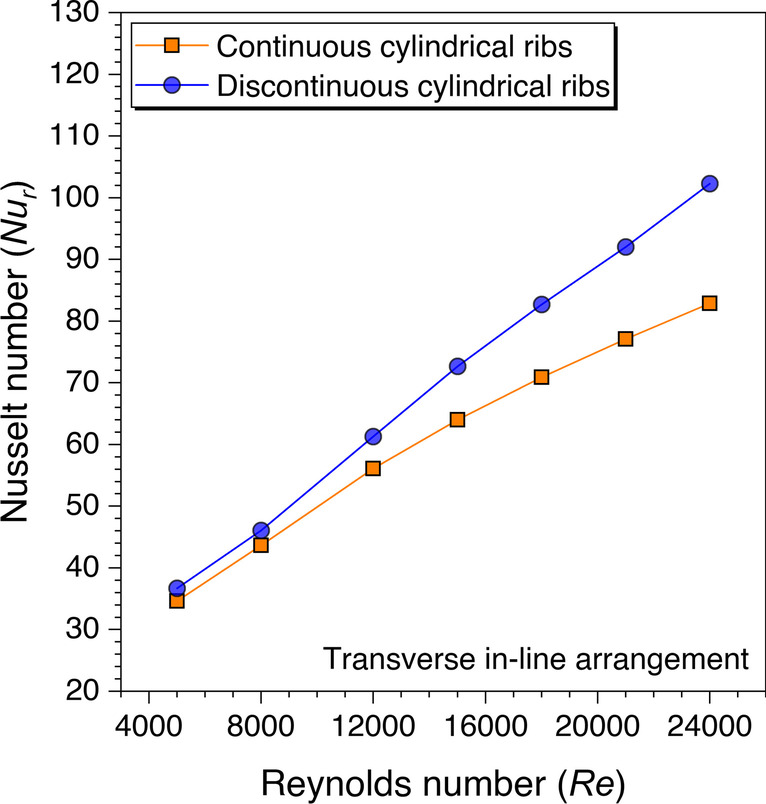
Variation of *Nu*_*r*_ against *Re* for cylindrical ribs

Similarly, the cylindrical ribs are also analyzed for the Nusselt number variation against the *Re* and presented in Fig. 21. It is observed that the discontinuous ribs perform better than the continuous ribs for considered *Re* ranges with cylindrical ribs. This is because of the nature of the continuous transverse cylindrical ribs as fluid particles form the re-circulation zone on either side of the ribs. Thus, these re-circulation zones store the heat and form the hotspots near the ribs, thereby, decreasing the heat transfer from the absorber plate to the flowing fluid. However, the discontinuities in the transverse cylindrical ribs make enough path for the stuck fluid to flow and mix with the primary fluid flow, resulting in better heat transfer from the absorber plate in comparison to the transverse continuous ribs. Hence, it is concluded that the discontinuous ribs perform better than the continuous ribs though the maximum value of the Nusselt number varies with the shape of the artificial ribs.

**Fig. 22:**
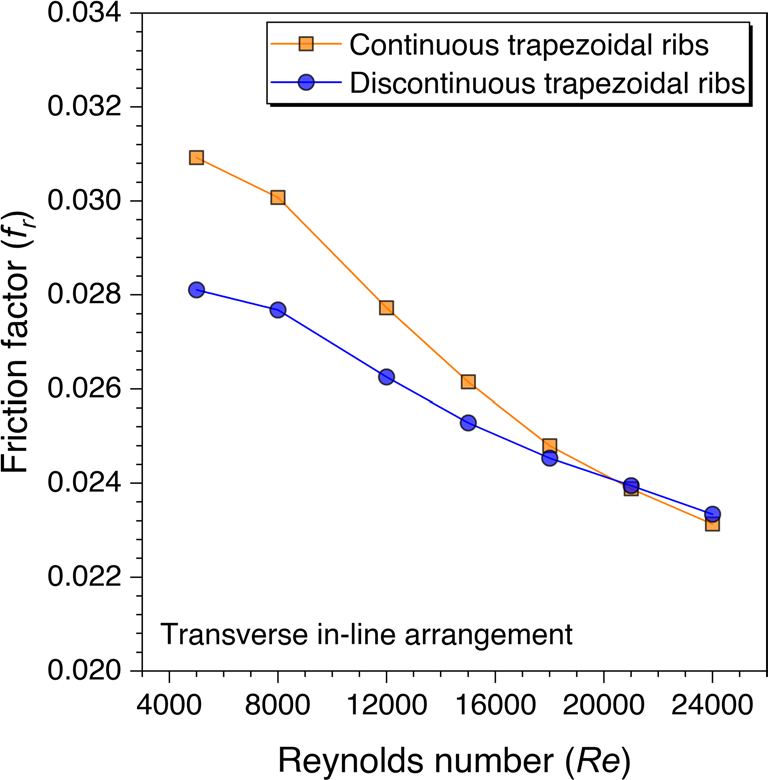
Variation of *f*_*r*_ against *Re* for trapezoidal ribs

After evaluating the heat transfer characteristics with the discontinuous ribs over continuous ribs, the friction factor behavior is also analyzed. Figure 22 shows the friction factor values variation against the Reynolds number for trapezoidal ribs. It is observed that the friction factor values decrease with increasing the Reynolds number values. This is because of subduing of the viscous sublayer on increasing *Re*. However, it is also observed that friction factor values are lower with discontinuous ribs in comparison to the continuous ribs. It is because the fluid particles are not restricted with that intensity as in the case of continuous ribs. It means fluid particles are getting enough space to move upstream without dropping the pressure that much to flow in the duct. However, the frictional factor values are found to be higher with the discontinuous ribs over continuous ribs after *Re* = 21,000 because the end-wall effects are prominent to showcase its effects in terms of the friction factor at a higher Reynolds number.

**Fig. 23:**
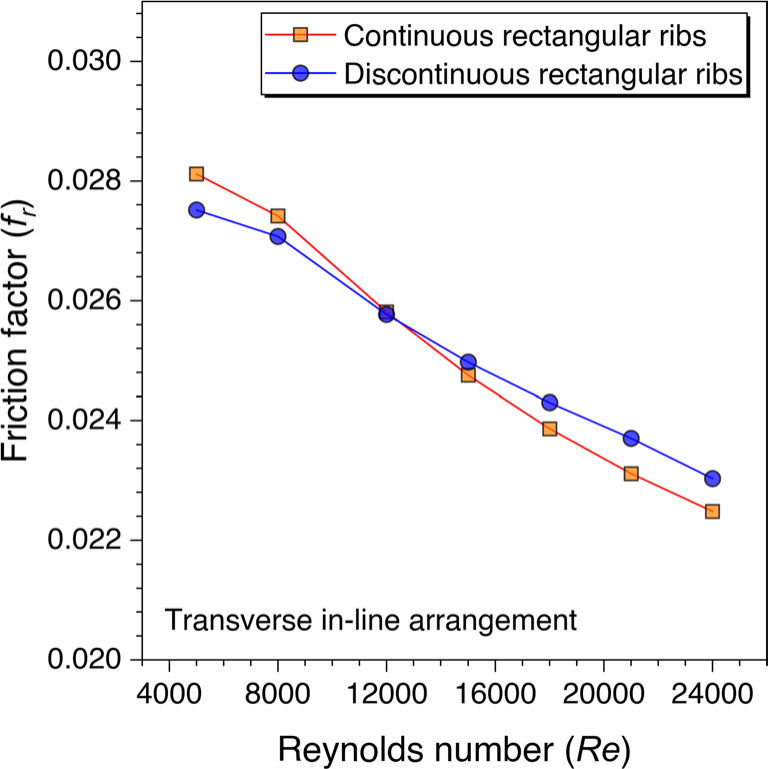
Variation of *f*_*r*_ against *Re* for rectangular ribs

Similarly, the friction factor variation against *Re* is also plotted for the discontinuous rectangular ribs in comparison to the continuous rectangular ribs, as shown in Fig. 23. It is observed that the friction factor values are observed to be lower with discontinuous ribs until *Re* = 12,000, after that the friction factor values with discontinuous ribs are found to be higher in comparison to the continuous ribs. This is because the sharp edges are more with the rectangular ribs in comparison to the trapezoidal ribs; thus, the sharp edges along with the end wall effects contribute to increasing the friction factor. Hence, it can be concluded that this trend was observed after *Re* = 21,000 with trapezoidal ribs, whereas this is found to be even after only *Re* = 12,000 with rectangular ribs, which means the sharp edges of any artificial roughness are not good at least for the friction factor. However, sharp edges impacts are only visible at the higher Reynolds number. Therefore, to quantify this argument, the cylindrical ribs are also investigated to see the impacts on the frictional factor characteristics.

**Fig. 24:**
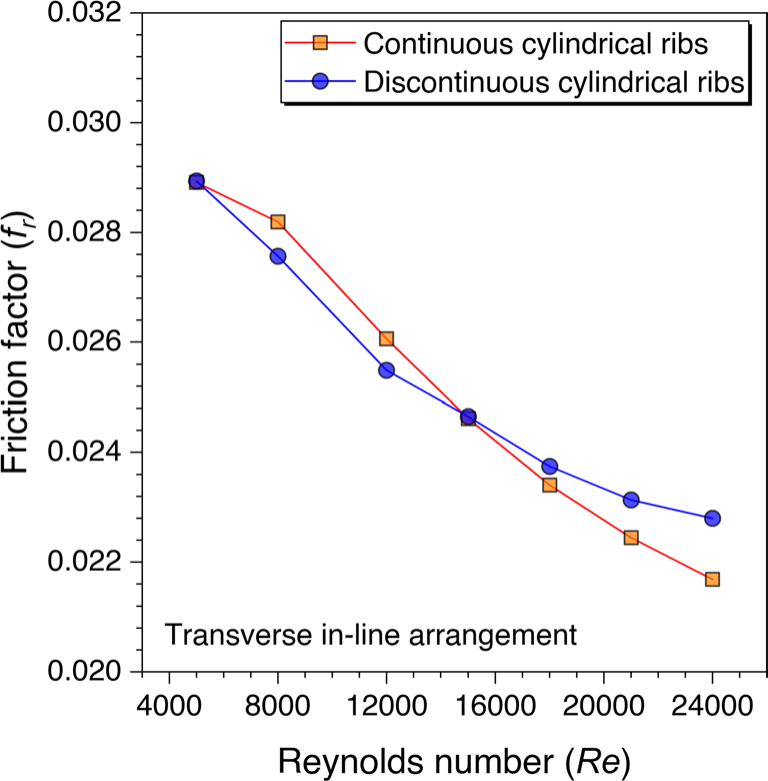
Variation of *f*_*r*_ against *Re* for cylindrical ribs

As stated in the last argument that the sharp edges do contribute to the friction factor values, however, these are observed at higher Reynolds numbers with discontinuous ribs. Therefore, the cylindrical ribs are also investigated and the variation of the friction factor values against *Re* is shown in Fig. 24. It is also observed with the cylindrical ribs that the friction factor values with discontinuous ribs are observed to be higher than the continuous ribs after *Re* = 15,000. Though the reason is stated in the discussion with the trapezoidal and rectangular ribs, here is also supported with the same reasoning that the sharp edges in the discontinuous ribs at higher Reynolds numbers are more visible.

### Thermal performance comparison of transverse ribs arranged in in-line and staggered configuration

**Fig. 25:**
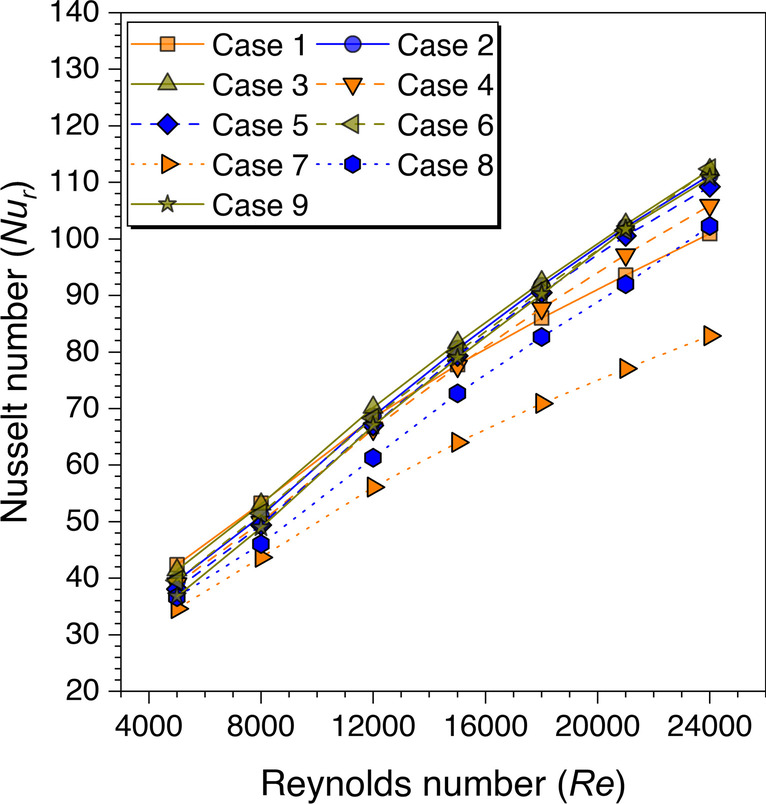
Variation of *Nu*_*r*_ against *Re* for ribs arranged in different configurations

Since the advantage of discontinuous ribs over continuous ribs is discussed in the preceding sections, therefore, the discontinuous ribs are now arranged in a staggered configuration. The aim is to understand the fluid flow behavior with staggered configuration over the in-line arrangement of the discontinuous ribs. Figure 25 shows the *Nu*_*r*_ values variation against *Re* for transverse continuous and discontinuous ribs arranged in in-line and staggered configurations. It is observed from Fig. 25 that the discontinuous ribs arranged in a staggered configuration perform better than the in-line configuration.

**Fig. 26:**
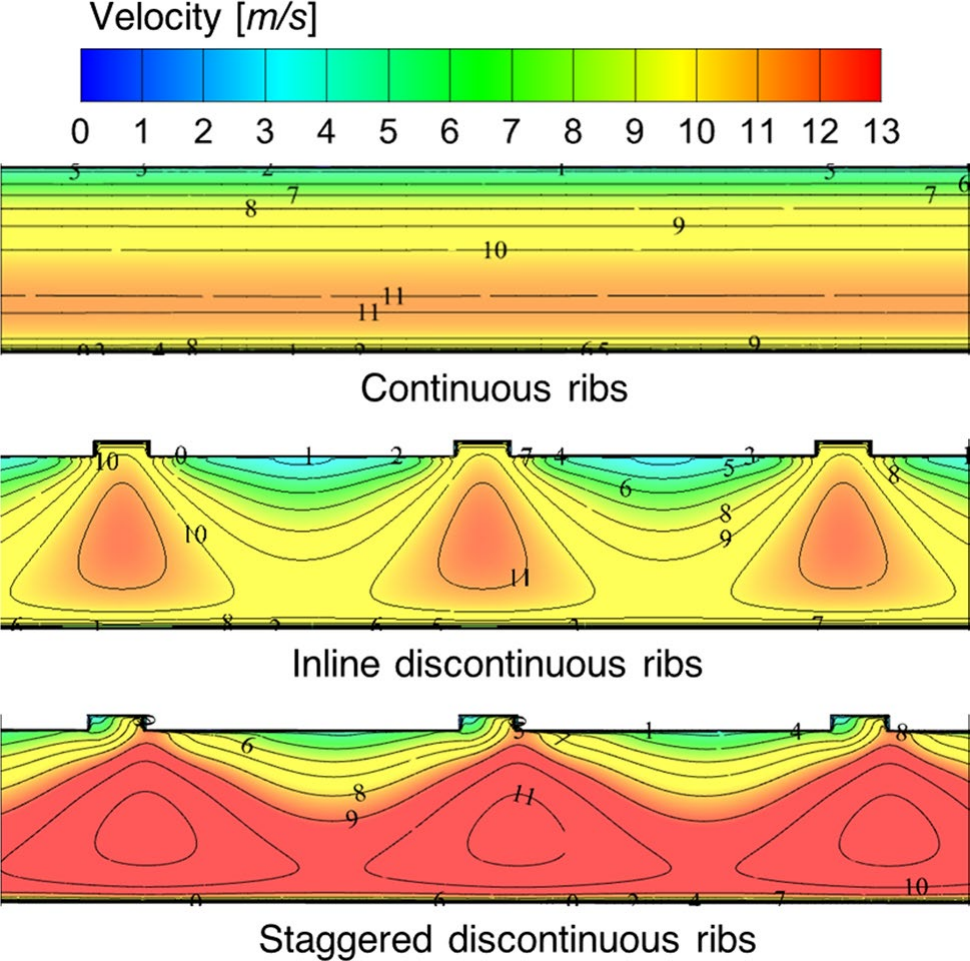
Velocity contours at a mid-plane in the transverse direction to the absorber plate

To understand the reason behind the Nusselt number values enhancement in staggered configuration arrangement of discontinuous ribs, the velocity contours are extracted at the mid-plane of the considered computational domain. The velocity contours for trapezoidal ribs are shown in Fig. 26. The velocity contours are shown for continuous and discontinuous ribs with in-line and staggered configurations. It is observed from the contours that the mixing of the fluid particles is increased in discontinuous ribs in comparison to the continuous ribs, and the high-velocity streams are moved to the center of the fluid domain, which was settled down near the lower wall of the test section with transverse continuous ribs. Further, more mixing is observed with a staggered arrangement compared to the in-line arrangement of the discontinuous ribs. Hence, it is concluded that the discontinuous ribs arranged with a staggered configuration are more effective because of the continuous mixing of the fluid particles due to their arrangement. These results are in agreement with the existing literature discussed in the literature survey (Cavallero and Tanda 2002; Tanda 2004a; Gill et al. 2017a).

**Fig. 27:**
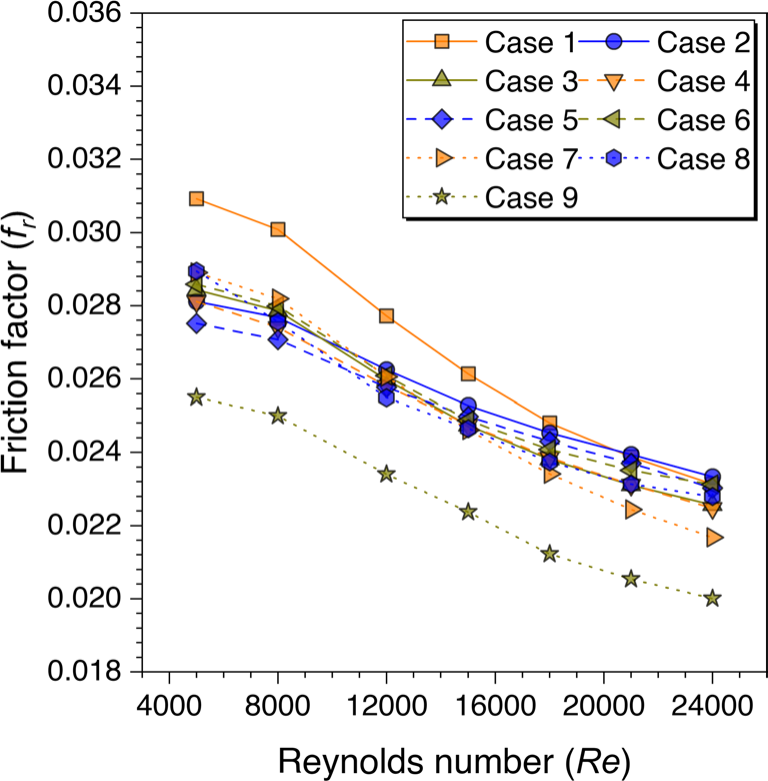
Variation of *f*_*r*_ with *Re* for ribs arranged in different configurations

In addition, the friction factor values variation against *Re* is plotted for all the cases and variation is shown in Fig. 27*.* The *f*_*r*_ values decrease on increasing the *Re* values for all the cases. This happened because of subduing the viscous sublayers with an increase in the Reynolds number. It is also observed from Fig. 27 that the *f*_*r*_ values are found maximum for transverse trapezoidal continuous ribs. In contrast, the values are minimum for cylindrical ribs arranged in a staggered configuration. However, the friction factor values for other cases are observed to be between the range of these two cases. The reason for this behavior is discussed in the preceding sections.

**Fig. 28:**
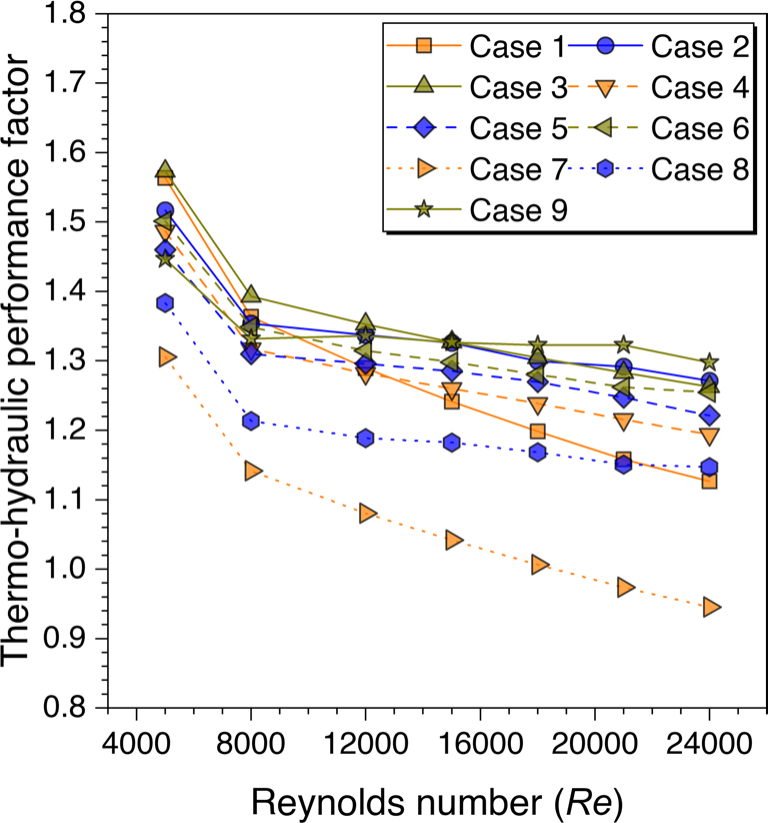
THPF variation against *Re* for ribs arranged in different configurations

Finally, the above results’ cumulative effects are plotted in terms of the variation of the THPF against *Re* for all the considered cases as shown in Fig. 28. The values are observed to be decreasing with increasing the *Re* values. However, the maximum THPF value is obtained with trapezoidal ribs arranged in staggered arrangement at *Re* = 5000 and this arrangement outperformed all other cases until *Re* = 15,000. After this Reynolds number value, the cylindrical ribs arranged in a staggered arrangement dominated all other arrangements of the ribs for the higher THPF value. Since it is concluded in the velocity contours discussion for the in-line and staggered arrangement that the fluid particles mix continuously as moving downstream in the duct, but this is not the case with the in-line arrangement as the fluid particles attain a certain path and do not mix as much in the staggered arrangement. Therefore, it is found that the ribs arranged in the staggered arrangement perform better in comparison to the other considered arrangement in the present study. Hence, the maximum value of THPF = 1.57 is obtained at *Re* = 5000 for the discontinuous trapezoidal ribs arranged in a staggered configuration. These results are in agreement with the existing literature discussed in the literature survey (Cavallero and Tanda 2002; Tanda 2004a; Gill et al. 2017a).

## Conclusions

An artificially roughened solar air heater is numerically investigated using CFD simulations to understand the thermal-fluid behavior. The absorber plate is artificially roughened with transverse ribs in a continuous and discontinuous manner. Discontinuous ribs are arranged in two different configurations, i.e., in-line and staggered. The effects of different cross-sections (viz., trapezoidal, circular, and square) of ribs are considered for thermal performance enhancement. The parametric optimization of discontinuous ribs is carried out for geometric parameters such as rib gap width (*g*) and discontinuous rib width (*w*) against the Reynolds number (*Re*). The range of *Re* is considered for investigation from 5000 to 24,000. It is observed that the value of *g* and *w* significantly influence the fluid flow and heat transfer characteristics. The results are reported in Nusselt number for heat transfer and friction factor for pressure drop. Both parameters are further calculated in terms of the thermo-hydraulic performance factor, which incorporates the combined effects of both parameters. The impacts of discontinuous ribs are compared with the continuous ribs having similar cross-sections. Hence, the following conclusions have been made based on the thorough CFD investigation:

1. The parametric optimization of rib gap width (*g*) in discontinuous ribs is carried out for values ranging from 5 to 12.5 mm. It is found that *g* = 10 mm is the optimum value for maximum heat transfer.
2. The parametric optimization of discontinuous rib width (*w*) in discontinuous ribs is conducted for values ranging from 20 to 50 mm. It is observed that *w* = 50 mm is the optimum value for maximum heat transfer.
3. Transverse discontinuous ribs have outperformed continuous transverse ribs in thermal performance enhancement.
4. Discontinuous ribs arranged in a staggered configuration are found to be the best performing. Trapezoidal discontinuous ribs arranged in a staggered configuration yielded the maximum thermo-hydraulic performance factor (THPF) equal to 1.57.
5. The Nusselt number (*Nu*_*r*_) values are observed to be rising upon increasing the Reynolds number values. In contrast, the friction factor (*f*_*r*_) values are found to be falling on increasing the Reynolds number. The maximum value of *Nu*_*r*_ and *f*_*r*_ is 112.3 for discontinuous rectangular ribs in staggered configuration and 0.031 for continuous trapezoidal ribs, respectively.

## Supplementary information

ESM 1 (DOCX 30 kb)

## Data Availability

The data supporting the findings of this study are available within the article.

## Nomenclature

*A*_*c*_: Rectangular inlet area [m^2^]
*A*_*s*_: Collector plate area [m^2^]
*C*_*p*_: Specific heat [J/kg-K]
*D*_*h*_: Hydraulic diameter [m]
*e*: Rib roughness height [m]
*g*: Gap width [m]
*h*: Convective heat transfer coefficient [W/m^2^-K]
*H*: Duct height [m]
*I*: Uniform heat flux [W/m^2^]
*k*: Thermal conductivity [W/m-K]
*L*: Length of the duct [m]
*P*: Longitudinal pitch length [m]
*P*_*wetted*_: Wetted perimeter [m]
*∆P*: Test section pressure drop [Pa]
*Q*: Total heat transfer [W]
*T*: Temperature [K]
*v*: Fluid velocity [m/s]
*w*: Discontinuous rib width [m]
*W*: Duct width [m]

## Dimensionless parameters

*e/D*_*h*_: Relative roughness height
*f*: Friction factor
*Nu*: Nusselt number
*P/e*: Relative roughness pitch
*Pr*: Prandtl number
*Re*: Reynolds number
*W/H*: Duct aspect ratio
*y*^*+*^: Dimensionless distance of the first cell from the collector plate

## Greek symbols

*k*: Turbulent kinetic energy [m^2^/s^2^]
*ε*: Dissipation rate [m^2^/s^3^]
*ρ*: Density [kg/m^3^]

## Subscripts

*i*: Inlet
*o*: Outlet
*w*: Wall
*r*: Roughened
*s*: Smooth

## Abbreviations

SAH: Solar air heater
CFD: Computational fluid dynamics
THPF: Thermo-hydraulic performance factor
TUI: Text user interface
ASHRAE: American Society of Heating, Refrigeration, and Air Conditioning Engineers
