# Supplementary material for: Optimization of a novel trapezoidal staggered ribs configuration for enhancement of a solar air heater performance using CFD
Source: Environ Sci Pollut Res Int. 2023 Jul 28;30(41):93582–601. doi: 10.1007/s11356-023-28978-9 (PMC10468433; doi:10.1007/s11356-023-28978-9)
Supplement: Supplementary file 1 — (DOCX 30 kb) [file 11356_2023_28978_MOESM1_ESM.docx]

**Appendix A.** Uncertainty assessment of the parameters used for experimental study.

The uncertainty in the experimental parameters is calculated based on the ANSI/ASME standard for measurement uncertainty, followed by the procedures of Kline and McClintock.

$\delta R= \pm\sqrt{[ \sum_{i=1}^{n} \left( \frac{\partial R}{{\partial x}_{i}}\delta x_{i} \right)^{2}]}$

The uncertainty contribution from each parameter is a product of uncertainty in the calculated parameter; $\delta x_{i}$ and the sensitivity coefficient; $\frac{\partial R}{{\partial x}_{i}}$. The procedure to calculate the uncertainty associated with each parameter is provided below based on the values given in ***Table A1***.

**Table A1.** The details of the parameters used for the experimental study

| **Measured parameter** | **Symbol** | **Value** | **Instrument** | **Least count of instruments** | **Uncertainty associated** |
| --- | --- | --- | --- | --- | --- |
| Absorber plate length | L_p_ | 450 mm | Linear scale | 1 mm | ± 1 mm |
| Diameter of the blower outlet | d | 75 mm | Vernier Caliper | 0.1 mm | ± 0.1 mm |
| Duct width | W | 300 mm | Linear scale | 1 mm | ± 1 mm |
| Duct height | H | 25 mm | Linear scale | 1 mm | ± 1 mm |
| Hydraulic diameter at the inlet | D_h_ | 46.154 mm | Calculated | - | ± 1.7 mm |
| Velocity at the blower outlet | v | 2.86 m/s | Anemometer | 0.01 m/s | ± 0.01 m/s |
| Mass flow rate at the outlet | $\dot{m}$ | 0.01472 Kg/s | Calculated | - | ± 0.00019 Kg/s |
| Specific heat of air | C_p_ | 1006.43 J/Kg-K | Constant | - | - |
| Inlet temperature of air | T_i_ | 28.60 K | Copper-constantan thermocouples | 0.01 K | ± 0.01 K |
| Outlet temperature of air | T_o_ | 38.60 K | Copper-constantan thermocouples | 0.01 K | ± 0.01 K |
| Total heat gain by fluid | Q_g_ | 148.15 W | Calculated | - | ± 2.6 W |
| Mean temperature of air | T_m_ | 33.60 K | Copper-constantan thermocouples | 0.01 K | ± 0.01 K |
| Wall temperature of absorber plate | T_w_ | 127.17 K | Copper-constantan thermocouples | 0.01 K | ± 0.01 K |
| Voltage | V | 150 V | Voltmeter | 0.1 V | ± 0.1 V |
| Current | I | 0.9 A | Ammeter | 0.1 A | ± 0.1 A |

**A.1.** The hydraulic diameter of the rectangular duct, D_h_

$$D_{h}= \frac{4WH}{2(W+H)}$$

$$\frac{\partial D_{h}}{\partial H}= \frac{2W}{(W+H)}- \frac{2WH}{{(W+H)}^{2}}$$

$$\frac{\partial D_{h}}{\partial H}= \frac{2 \times300}{(300+25)}- \frac{2\times300\times25}{\left( 300+25 \right)^{2}} =1.704$$

And,

$$\frac{\partial D_{h}}{\partial W}= \frac{2H}{(W+H)}- \frac{2WH}{{(W+H)}^{2}}$$

$$\frac{\partial D_{h}}{\partial W}= \frac{2 \times25}{(300+25)}- \frac{2\times300\times25}{\left( 300+25 \right)^{2}} =0.012$$

$$\delta D_{h}=\left[ \left\{ \frac{\partial D_{h}}{\partial W}\delta W \right\}^{2}+\left\{ \frac{\partial D_{h}}{\partial H}\delta H \right\}^{2} \right]^{0.5}$$

$$\delta D_{h}=\left[ \left\{ 0.012\times1 \right\}^{2}+\left\{ 1.704\times1 \right\}^{2} \right]^{0.5}= \pm1.704 mm$$

Hence, $\frac{\delta D_{h}}{D_{h}}=0.037=3.7 \%$

**A.2.** The cross-sectional area of the rectangular duct, A_c_

$$A_{c}= W\times H$$

$$\frac{\partial A_{c}}{\partial H}= W$$

$$\frac{\partial A_{c}}{\partial H}= 300$$

And,

$$\frac{\partial A_{c}}{\partial W}= H$$

$$\frac{\partial A_{c}}{\partial W}= 25$$

$$\delta A_{c}=\left[ \left\{ \frac{\partial A_{c}}{\partial W}\delta W \right\}^{2}+\left\{ \frac{\partial A_{c}}{\partial H}\delta H \right\}^{2} \right]^{0.5}$$

$\delta A_{c}=\left[ \left\{ 25\times1 \right\}^{2}+\left\{ 300\times1 \right\}^{2} \right]^{0.5}= \pm301.04 mm$^2^

Hence, $\frac{\delta A_{c}}{A_{c}}=0.04014=4.014 \%$

**A.3.** The absorber plate area, A_p_

$$A_{p}= W\times L_{p}$$

$$\frac{\partial A_{p}}{\partial L_{p}}= W$$

$$\frac{\partial A_{p}}{\partial L_{p}}= 300$$

And,

$$\frac{\partial A_{p}}{\partial W}= L_{p}$$

$$\frac{\partial A_{p}}{\partial W}= 450$$

$$\delta A_{p}=\left[ \left\{ \frac{\partial A_{p}}{\partial W}\delta W \right\}^{2}+\left\{ \frac{\partial A_{p}}{\partial L_{p}}\delta L_{p} \right\}^{2} \right]^{0.5}$$

$\delta A_{p}=\left[ \left\{ 450\times1 \right\}^{2}+\left\{ 300\times1 \right\}^{2} \right]^{0.5}= \pm540.83 mm$^2^

Hence, $\frac{\delta A_{p}}{A_{p}}=0.00401=0.401 \%$

**A.4.** Mass flow rate at outlet of centrifugal blower, $\dot{m}$

$$\dot{m}=\rho A_{c,blower}V$$

As the thermo-physical properties are assumed to be constant because negligible change is observed at the mean fluid temperature of the flowing fluid,

$$A_{c,blower}= \frac{\pi}{4} d^{2}$$

$$\frac{\partial A_{c,blower}}{\partial d}= \frac{\pi}{2}d$$

$$\delta A_{c,blower}= \left[ \left\{ \frac{\partial A_{c,blower}}{\partial d}\delta d \right\}^{2} \right]^{0.5}$$

$\delta A_{c,blower}= \left[ \left\{ \frac{\pi}{2}d \delta d \right\}^{2} \right]^{0.5}= \frac{\pi}{2}d \delta d=\frac{3.14}{2} \times75\times0.5= \pm58.875 mm$^2^

Hence, $\frac{\delta A_{c,blower}}{A_{c,blower}}=0.01333=1.33 \%$

$$\frac{\partial\dot{m}}{\partial A_{c,blower}}= \rho V$$

$$\frac{\partial\dot{m}}{\partial A_{c,blower}}= \rho\times2.86$$

$$\frac{\partial\dot{m}}{\partial V}= \rho A_{c,blower}$$

$$\frac{\partial\dot{m}}{\partial V}= \rho\times0.00442$$

$$\delta\dot{m}=\left[ \left\{ \frac{\partial\dot{m}}{\partial A_{c,blower}}{\delta A}_{c,blower} \right\}^{2}+\left\{ \frac{\partial\dot{m}}{\partial V}\delta V \right\}^{2} \right]^{0.5}$$

$$\delta\dot{m}{=\left[ \left\{ \rho\times2.86\times58.875\times{10}^{-6} \right\}^{2}+\left\{ \rho\times0.00442 \times0.01 \right\}^{2} \right]}^{0.5}$$

$\delta\dot{m}=\rho\times$0.0001741

$$\frac{\delta\dot{m}}{\dot{m}}=0.0143=1.43 \%$$

**A.5.** Reynolds number, Re

$$Re= \frac{\rho vD_{h}}{\mu}$$

$$\frac{\delta Re}{Re}= \left[ \left\{ \frac{\delta v}{v} \right\}^{2}+\left\{ \frac{\delta D_{h}}{D_{h}} \right\}^{2} \right]^{0.5}$$

$$\frac{\delta Re}{Re}= \left[ \left\{ \frac{0.01}{2.86} \right\}^{2}+\left\{ 0.037 \right\}^{2} \right]^{0.5}=0.0372=3.72 \%$$

**A.6.** Total heat gain by the fluid flow, $Q_{g}$

$$Q_{g}= \dot{m}C_{p}\left( T_{o}- T_{i} \right)$$

As the specific heat is assumed to be constant throughout the analysis, therefore,

$$\frac{\delta Q_{g}}{Q_{g}}= \left[ \left\{ \frac{\delta\dot{m}}{\dot{m}} \right\}^{2}+\left\{ \frac{\delta\Delta T}{\Delta T} \right\}^{2} \right]^{0.5}$$

$$\frac{\delta Q_{g}}{Q_{g}}= \left[ \left\{ 0.0143 \right\}^{2}+\left\{ \frac{0.1}{10} \right\}^{2} \right]^{0.5}=0.0174=1.74 \%$$

**A.7.** Convective heat transfer coefficient, h

$$h= \frac{Q_{g}}{A_{p}(T_{w}-T_{m})}= \frac{Q_{g}}{A_{p}(\Delta T_{wm})}$$

$$\frac{\delta h}{h}= \left[ \left\{ \frac{\delta Q_{g}}{Q_{g}} \right\}^{2}+\left\{ \frac{\delta A_{p}}{A_{p}} \right\}^{2}+\left\{ \frac{\delta\Delta T_{wm}}{\Delta T_{wm}} \right\}^{2} \right]^{0.5}$$

$$\frac{\delta h}{h}= \left[ \left\{ 0.0174 \right\}^{2}+\left\{ 0.00411 \right\}^{2}+\left\{ \frac{0.1}{70.98} \right\}^{2} \right]^{0.5}=0.0179=1.79 \%$$

**A.8.** Nusselt number, Nu

$$Nu= \frac{hD_{h}}{k_{air}}$$

$$\frac{\delta Nu}{Nu}= \left[ \left\{ \frac{\delta h}{h} \right\}^{2}+\left\{ \frac{\delta D_{h}}{D_{h}} \right\}^{2} \right]^{0.5}$$

$$\frac{\delta Nu}{Nu}= \left[ \left\{ 0.018 \right\}^{2}+\left\{ 0.037 \right\}^{2} \right]^{0.5}=0.0411=4.11 \%$$

The ranges of the uncertainty assessment calculated for all the experimental investigated Reynolds number are provided in ***Table A2***.

**Table A2.** Ranges of associated uncertainty with calculated parameters

| **Parameters** | **Smooth duct** | |
| --- | --- | --- |
|  | **Values** | **Uncertainty range (%)** |
| Mass flow rate, $\dot{m}$ [Kg/s] | 0.01472–0.060012 | 1.43–1.33 |
| Reynolds number, *Re* | 4879.07–19891.51 | 3.72–3.70 |
| Convective heat transfer coefficient, *h* [W/m^2^K] | 11.73–33.05 | 1.79–6.84 |
| Nusselt number, *Nu* | 20.51–57.78 | 4.11–7.77 |
